# Inhibition of EHMT1/2 rescues synaptic damage and motor impairment in a PD mouse model

**DOI:** 10.1007/s00018-024-05176-5

**Published:** 2024-03-12

**Authors:** Zhixiong Zhang, Rui Wang, Hui Zhou, Dan Wu, Yifan Cao, Chuang Zhang, Hongyang Sun, Chenchen Mu, Zongbing Hao, Haigang Ren, Nana Wang, Shuang Yu, Jingzhong Zhang, Mengdan Tao, Can Wang, Yan Liu, Liu Liu, Yanli Liu, Jianye Zang, Guanghui Wang

**Affiliations:** 1 Laboratory of Molecular Neuropathology, Department of Pharmacology, Jiangsu Key Laboratory of Neuropsychiatric Diseases and College of Pharmaceutical Sciences, Soochow University, Suzhou, 215123 Jiangsu China; 2 Jiangsu Provincial Medical Innovation Center of Trauma Medicine, Institute of Trauma Medicine, Soochow University, Suzhou, 215123 Jiangsu China; 3 MOE Key Laboratory of Geriatric Diseases and Immunology, Soochow University, Suzhou, 215123 Jiangsu China; 4 Suzhou Institute of Biomedical Engineering and Technology, Chinese Academy of Sciences, Suzhou, 215163 China; 5 School of Pharmacy, Collaborative Innovation Center for Cardiovascular Disease Translational Medicine, State Key Laboratory of Reproductive Medicine, Nanjing Medical University, Nanjing, 211166 China; 6 Department of Pharmacy, The First Affiliated Hospital of Hunan Normal University, Hunan Provincial People’s Hospital, Changsha, 410005 China; 7 College of Pharmaceutical Sciences, Soochow University, Suzhou, 215123 Jiangsu China; 8 Hefei National Laboratory for Physical Sciences at Microscale CAS Center for Excellence in Biomacromolecules, Collaborative Innovation Center of Chemistry for Life Sciences, and School of Life Sciences, University of Science and Technology of China, 96 Jinzhai Road, Hefei, 230026 Anhui China; 9 Center of Translational Medicine, First People’s Hospital of Taicang, Taicang Affiliated Hospital of Soochow University, Suzhou, 215400 China

**Keywords:** Parkinson’s disease, α-synuclein preformed fibrils, Histone H3 dimethylation, Synaptic dysfunction, Motor impairment

## Abstract

Epigenetic dysregulation that leads to alterations in gene expression and is suggested to be one of the key pathophysiological factors of Parkinson’s disease (PD). Here, we found that α-synuclein preformed fibrils (PFFs) induced histone H3 dimethylation at lysine 9 (H3K9me2) and increased the euchromatic histone methyltransferases EHMT1 and EHMT2, which were accompanied by neuronal synaptic damage, including loss of synapses and diminished expression levels of synaptic-related proteins. Furthermore, the levels of H3K9me2 at promoters in genes that encode the synaptic-related proteins SNAP25, PSD95, Synapsin 1 and vGLUT1 were increased in primary neurons after PFF treatment, which suggests a linkage between H3K9 dimethylation and synaptic dysfunction. Inhibition of EHMT1/2 with the specific inhibitor A-366 or shRNA suppressed histone methylation and alleviated synaptic damage in primary neurons that were treated with PFFs. In addition, the synaptic damage and motor impairment in mice that were injected with PFFs were repressed by treatment with the EHMT1/2 inhibitor A-366. Thus, our findings reveal the role of histone H3 modification by EHMT1/2 in synaptic damage and motor impairment in a PFF animal model, suggesting the involvement of epigenetic dysregulation in PD pathogenesis.

**Supplementary Information:**

The online version contains supplementary material available at 10.1007/s00018-024-05176-5.

## Introduction

Parkinson’s disease (PD) is the second most common neurodegenerative disorder characterized by progressive loss of striatal-projecting dopaminergic (DA) neurons in the substantia nigra (SN) and the occurrence of misfolding and intracellular aggregation of α-synuclein (α-syn), known as Lewy bodies (LBs) [1–3]. However, the etiology of PD is incompletely understood, although many genes and multiple pathways have been found to be associated with PD pathogenesis [4–8].

The aggregation of α-syn is the main pathological feature of PD. Overexpression of α-syn or treatment with α-synuclein preformed fibrils (PFFs) results in a defect in synaptic vesicle recycling, markedly inhibits neurotransmitter release [9] or compromises the colocalization of synaptic markers and dendritic spine dynamics to maintain synaptic activity [10]. As α-syn is a causative factor in PD and PFFs have broad effects on multiple cellular processes, α-syn may trigger some upstream effectors that result in alterations in multiple pathways. During the formation of Lewy bodies, mitochondrial damage and synaptic dysfunction occur in the PFF model in vitro [11], ultimately leading to neuronal death [12]. Furthermore, in α-syn-overexpressing or PFF-seeded model mice, decreased spine density and abnormalities in spine dynamics were shown [13], and the accumulation of α-syn significantly exacerbates synaptic damage and neuronal degeneration in vivo [14].

Synaptic dysfunction is considered to be an early event, followed by the active deconstruction of axons and loss of neuronal connectivity in the dopaminergic degeneration associated with PD, as the loss of striatal dopaminergic terminals seems to precede neuronal loss in the substantia nigra pars compacta (SNpc) [15–18]. A recent study identified a pathway for the selective activation of parkin at human dopaminergic synapses and revealed that synaptic dysfunction may represent an initial pathogenic event in this disease [19]. In the early stages of PD patients, positron emission tomography (PET) reveals extensive axonal damage and loss of connectivity in the nigrostriatal pathway [20]. However, the mechanism underlying the α-syn pathology and synaptic damage in PD, as well as eventual neuronal death, have not been elucidated.

Recently, epigenetic modifications, such as DNA methylation [21, 22], histone acetylation [23, 24] and miRNA [25], have been suggested to contribute to disease progression in PD patients. Histone methylation is associated with either transcriptional repression or activation. Histone methylation is involved in the dysregulation of synaptic functions in association with mental disorders [26, 27]. In recent years, many studies have implicated epigenetic regulation, especially histone H3 methylation, in synaptic functions from neurogenesis to neurodegenerative disease [28–30]. Dimethylation at lysine 9 of histone H3 (H3K9me2) is associated with transcriptional gene repression, which depends on the H3K9-specific histone methyltransferase EHMT1/2 (also named GLP/G9a) [31, 32]. Specific inhibition of EHMT1/2 rescues autism-like social deficits in Shank3-deficient mice and restores NMDAR-mediated synaptic function [33]. Furthermore, neuron-specific deficiency of EHMT1/2 leads to defects in learning, motivation and environmental adaptation in mice, similar to the key symptoms of Kleefstra syndrome [34, 35]. In addition, the expression levels of EHMT1 in the prefrontal cortex correlate positively with the progression of Alzheimer’s disease (AD), and inhibition of EHMT1/2 rescues synaptic and cognitive functions in AD model mice [36, 37]. These studies suggest the involvement of histone methylation by EHMT1/2 in the regulation of synaptic functions and the progression of diseases. However, whether pathological connections occur between α-syn pathology and EHMT1/2 and how they influence synaptic functions in PD remain largely unknown.

In this study, we revealed the role of EHMT1/2 in histone methylation in PD pathophysiology. We demonstrated that PFFs increase EHMT1/2 expression, leading to the upregulation of H3K9me2, which induces a decrease in the expression of synaptic-related proteins. Moreover, H3K9me2 was enriched at the promoters of *SNAP25*, *PSD95*, *Synapsin 1* and *vGLUT1* after PFF treatment. Furthermore, inhibition of EHMT1/2 with the inhibitor A-366 or shRNA significantly restored synaptic protein expression in primary neurons, and A-366 rescued motor impairments in PFF-treated mice. Thus, our study suggested the involvement of H3K9me2 modification by EHMT1/2 in synaptic dysfunction in a PD model.

## Results

### PFFs cause increases in H3K9me2 histone methylation and decreases in synapse-associated proteins

**Fig. 1:**
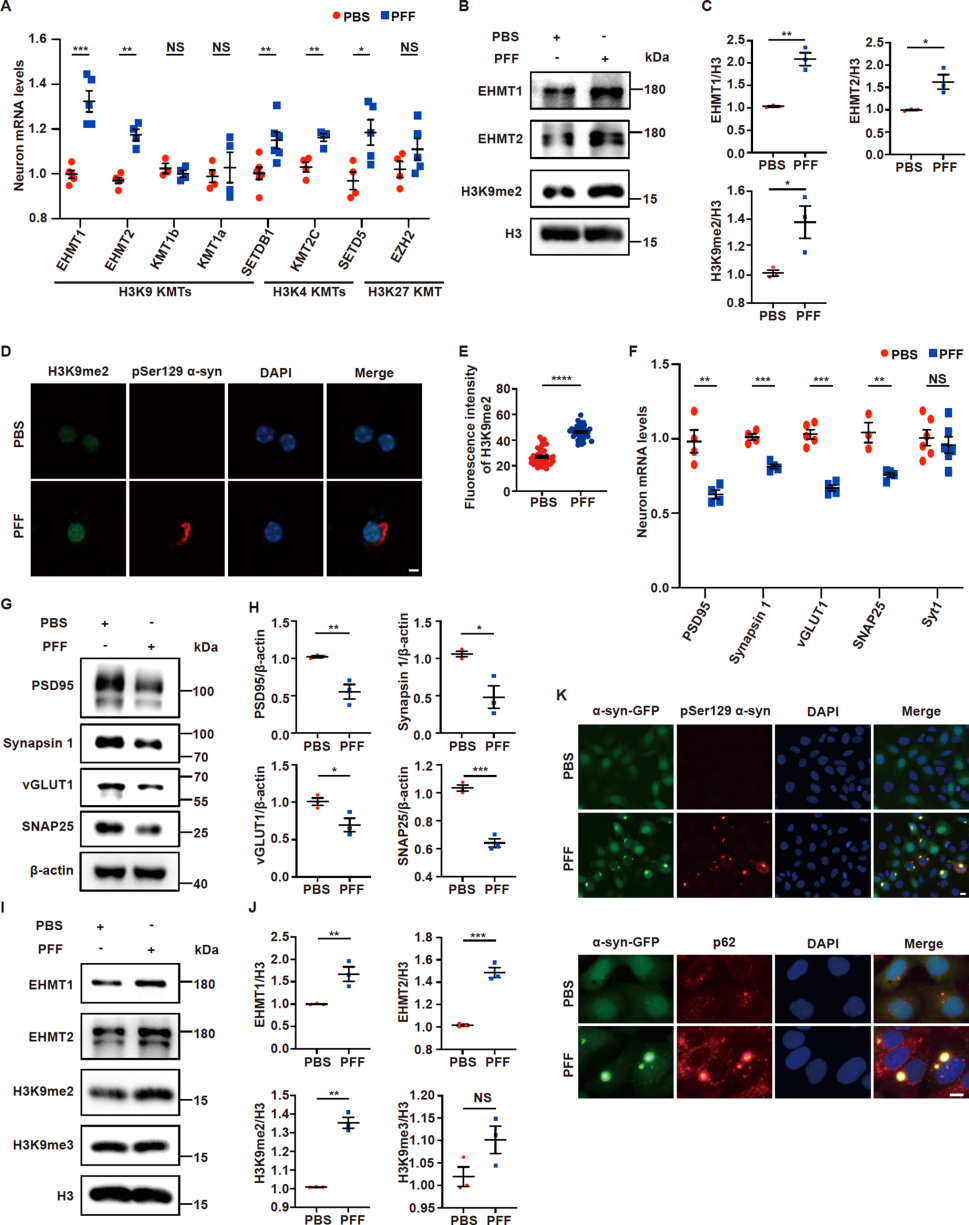
PFFs cause increases in H3K9me2 histone methylation and decreases in synapse-associated proteins. (**A**–**H**) Primary neurons were incubated with 3 µg/mL PFFs at 7 DIV for 14 days and then processed for analysis. **A** Quantitative real-time PCR data showing the mRNA levels of H3K9, H3K27 and H3K4 methyltransferases in primary neurons treated with PFFs or PBS (n = 3–5). **B**–**C** Immunoblots and quantitative analysis of EHMT1, EHMT2 and H3K9me2 protein levels in primary neurons (n = 3). **D** Representative confocal images of H3K9me2 and pSer129 α-syn immunofluorescence staining in primary neurons. Scale bar, 10 μm. **E** Plots showing the level of H3K9me2 (n = 40). **F** Quantitative real-time PCR data showing the mRNA levels of *PSD95*, *Synapsin 1*, *vGLUT1*, *SNAP25* and *Syt1* in primary neurons treated with PFFs or PBS (n = 3–6). **G**–**H** Immunoblots and quantitative analyses of PSD95, Synapsin 1, vGLUT1 and SNAP25 protein levels in primary neurons (n = 3). (**I**–**K**) PFFs were transfected into HEK293-α-syn-GFP cells at 3 µg/mL for 72 h and then processed for analysis. **I**–**J** Immunoblots and quantitative analysis of EHMT1, EHMT2, H3K9me2 and H3K9me3 protein levels in the HEK293-α-syn-GFP stable cell line treated with PFFs or PBS (n = 3). **K** Representative images of immunofluorescence staining of pSer129 α-syn, p62 and α-syn-GFP in HEK293-α-syn-GFP cells. Scale bar, 10 μm. The *P* values were calculated using an unpaired t test in **A**, **C**, **E**, **F**, **H** and **J**. *P < 0.05, **P < 0.01, ***P < 0.001, ****P < 0.0001, NS indicates not significant. The data are represented as the mean ± SEM

To determine whether α-syn alters histone methylation, we first prepared PFFs using recombinant α-syn, identified the oligomerization of α-syn using western blotting and examined the effects of PFFs on primary neurons (Fig. S1A–D), revealing the formation of fibrils, oligomerization of α-syn and induction of α-syn pathology. Next, we treated primary neurons with PFFs and examined the expression of several sets of genes that are associated with H3 methylation. Several H3K9 and H3K4 histone methyltransferases were upregulated upon PFF treatment (Fig. 1A). We selected the H3K9-specific histone methyltransferases EHMT1 and EHMT2 for further analyses because it has been reported that *EHMT2* is associated with PD in European and East Asian populations [38]. In PFF-treated primary neurons, the expression levels of both EHMT1 and EHMT2 were increased (Fig. 1B, C). Furthermore, H3K9me2, which is specifically methylated by EHMT1 and EHMT2, was increased (Fig. 1B, C). In addition, H3K9me2 staining increased in PFF-treated primary neurons that were positively labeled with the pSer129 α-syn antibody (Fig. 1D, E). As H3K9me2 is transcriptionally repressive and because the activity of its methyltransferases EHMT1 and EHMT2 is associated with synaptic functions [37, 39], we examined synapse-related gene expression. In PFF-treated neurons, the expression levels of the synapse-related genes *PSD95*, *Synapsin1*, *vGLUT1* and *SNAP25* were decreased, but not that of *Syt1* (Fig. 1F). Biochemical analyses also revealed that the expression of PSD95, Synapsin 1, vGLUT1 and SNAP25 in primary neurons decreased after PFF treatment (Fig. 1G, H).

To explore the role of α-syn pathology and histone methylation, we also treated the HEK293-α-syn-GFP cell line that stably expresses α-syn-GFP with PFFs. PFFs significantly induced the oligomerization of α-syn-GFP in the HEK293-α-syn-GFP cell line (Fig. S2A). Like in PFF-treated primary neurons, in the HEK293-α-syn-GFP stable cell line treated with PFFs, the levels of H3K9me2 but not H3K9me3 were significantly increased (Fig. 1I, J). Furthermore, the expression levels of both EHMT1 and EHMT2 were increased (Fig. 1I, J). In addition, immunofluorescence analyses revealed that PFFs induced α-syn-GFP to form pathological α-syn, which was labeled with the pSer129 antibody (Fig. 1K). Moreover, pSer129 α-syn colocalized with p62 (Fig. 1K).

In HEK293 cells, neither the overexpression of α-syn-GFP nor treatment with PFFs alone induced the formation of α-syn oligomers due to the lack of endogenous α-syn in HEK293 cells (Fig. S2B). We next wanted to confirm whether the alterations in epigenetic regulation depend on the formation of α-syn oligomers. We transfected HEK293 cells with GFP-N1 or α-syn-GFP or treated HEK293 cells with PFFs for 48 h. No changes in the protein levels of H3K9me3 or H3K9me2 were observed (Fig. S2C), suggesting an association between epigenetic changes and the formation of α-syn oligomers.

### Inhibition of EHMT1/2 restores the expression levels of synapse-related proteins

To further identify the mediation of EHMT1/2 in PFF-induced H3K9me2 modification, we treated the HEK293-α-syn-GFP stable cell line with the EHMT1/2 inhibitor A-366 at the indicated concentrations (Fig. S3A). We found that A-366 did not affect the protein levels of EHMT1 or EHMT2 (Fig. S3A). We used an appropriate concentration of A-366 to treat stable HEK293-α-syn-GFP cells after 24 h of PFF treatment (Fig. S3B). The protein levels of H3K9me2 were significantly increased upon PFF treatment (Fig. S3B). However, A-366 treatment significantly blocked the PFF-induced increase in H3K9me2 levels (Fig. S3B). In addition, neither H3K9me3 nor H3K4me3 was altered by PFFs or A-366. These data suggest that PFFs have specific effects on H3K9me2 and that A-366 specifically inhibits H3K9me2.

**Fig. 2:**
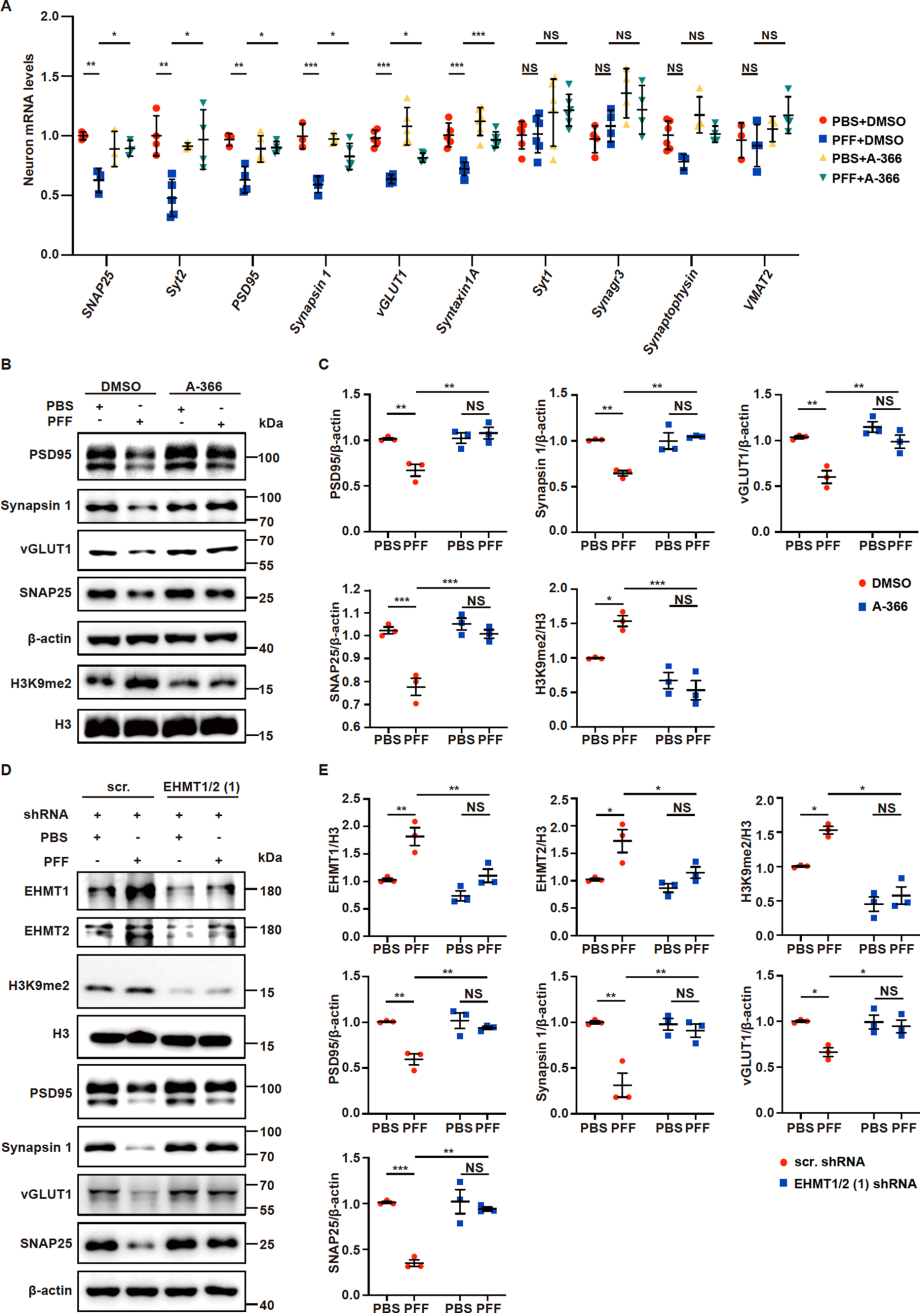
Inhibition of EHMT1/2 rescues the decline in synapse-related proteins by reducing the level of H3K9me2. (**A**–**C**) Primary neurons were incubated with 3 µg/mL PFFs at 7 DIV for 14 days and then processed for analysis. Treatment with A-366 was administered according to the schematic diagram depicted in Supple Fig. 3C. **A** Quantitative real-time PCR data showing the mRNA levels of *SNAP25*, *Syt2*, *PSD95*, *Synapsin 1*, *vGLUT1*, *Syntaxin1A*, *Syt1*, *Synagr3*, *Synaptophysin* and *VMAT2* in primary neurons (n = 3–5). **B**–**C** Immunoblots and quantitative analysis of PSD95, Synapsin 1, vGLUT1, SNAP25 and H3K9me2 (n = 3). **D**–**E** Immunoblots and quantitative analysis of EHMT1, EHMT2, H3K9me2, PSD95, Synapsin 1, vGLUT1, and SNAP25 in primary neurons treated with PFFs, or PBS and scr. shRNA or EHMT1/2 (1) shRNA (n = 3). shRNAs were added to primary neurons at 5 DIV. Subsequently, PFFs were added to primary neurons at 7 DIV for 14 days. The *P* values were calculated using two-way ANOVA in **A**, **C** and **E**. *P < 0.05, **P < 0.01, ***P < 0.001, NS indicates not significant. Data are represented as the mean ± SEM

We showed that PFF treatment decreases the expression of synaptic proteins (Fig. 1). We wondered whether the effects of PFFs on the expression of synaptic proteins are associated with EHMT1/2. We examined the expression levels of synaptic proteins in primary neurons treated with PFFs alone or in combination with A-366. Primary neurons were cultured for 7 days in vitro (DIV) and then exposed to PFFs. A-366 was added to cultured neurons at a dose of 2 µM at 9 DIV and added every 72 h for 12 days, according to the literature [40] (Fig. S3C). We detected the mRNA levels of several synaptic-related genes in primary neurons treated with PFFs alone or in combination with A-366. Among them, the mRNA levels of the synapse-related genes *SNAP25*, *Synapsin 1*, *Syt2*, *PSD95*, *vGLUT1* and *Syntaxin1A* were significantly decreased; however, A-366 treatment attenuated the PFF-induced decreases in the expression of *SNAP25*, *Synapsin1*, *Syt2*, *PSD95*, *vGLUT1* and *Syntaxin1A* (Fig. 2A). Moreover, the protein levels of PSD95, Synapsin 1, vGLUT1 and SNAP25 were decreased in PFF-treated primary neurons (Fig. 2B, C). Moreover, H3K9me2 was increased upon PFF treatment (Fig. 2B, C). However, A-366 treatment restored the levels of synaptic proteins that were impaired by PFFs and blocked the increase in H3K9me2 caused by PFFs (Fig. 2B, C). Given the limitations of pharmacological agents in inhibiting histone methyltransferases, we performed RNA interference (RNAi)-mediated knockdown of *EHMT1* and *EHMT2* to examine the effects of these agents on the expression of synaptic proteins in primary neurons. The protein levels of EHMT1 and EHMT2 were significantly decreased after neurons were infected with shRNA lentiviruses against *EHMT1* and *EHMT2* (Figs. 2D, E and S3D, E). Moreover, the PFF-induced increase in H3K9me2 levels was significantly inhibited by shRNAs targeting *EHMT1* and *EHMT2* (Figs. 2D, E and S3D, E). Furthermore, knockdown of *EHMT1* and *EHMT2* significantly restored the protein levels of PSD95, Synapsin 1, vGLUT1, and SNAP25, which were downregulated by PFFs (Figs. 2D, E and S3D, E).

**Fig. 3:**
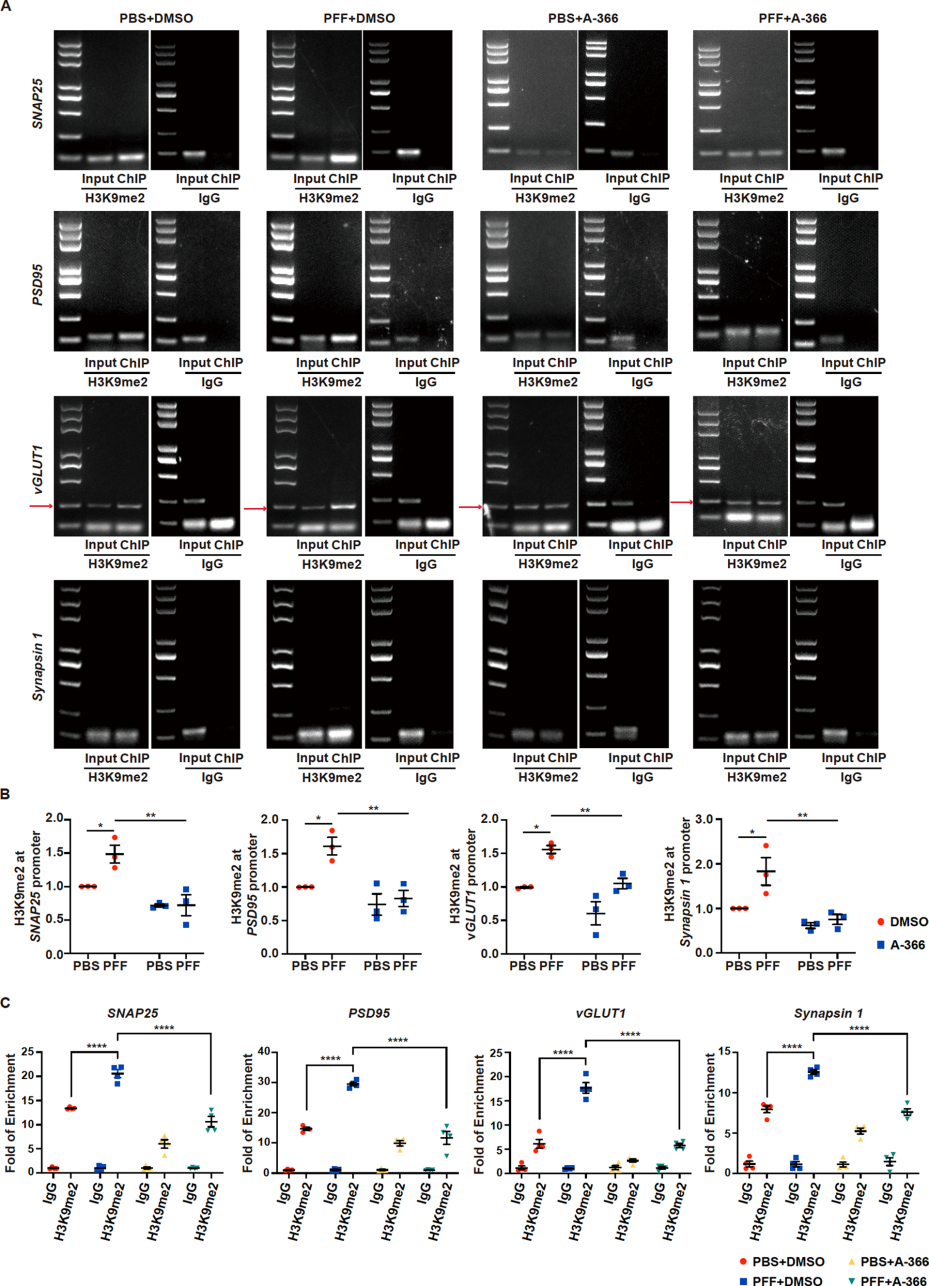
H3K9me2 methylation at gene promoters in primary neurons treated with PFFs or PBS or A-366 or DMSO. Primary neurons were incubated with 3 µg/mL PFFs at 7 DIV for 14 days and then processed for analysis. Treatment with A-366 was administered according to the schematic diagram depicted in Supple Fig. 3C. **A** PCR images showing the input (total DNA) and ChIP (H3K9me2-occupied DNA) signals at the upstream transcription start site regions of *SNAP25*, *PSD95*, *vGLUT1* and *Synapsin 1*. Mouse IgG was used as the negative control. **B** ChIP assay data showing the enrichment of H3K9me2 at the *SNAP25*, *PSD95*, *vGLUT1* and *Synapsin 1* promoter regions. (n = 3). **C** ChIP‒qPCR data comparing the enrichment of H3K9me2 at *SNAP25*, *PSD95*, *vGLUT1* and *Synapsin 1* in neurons treated with PFFs or PBS and A-366 or DMSO. Mouse IgG was used as the negative control. (n = 4). The *P* values were calculated using two-way ANOVA in **B** and **C**. *P < 0.05, **P < 0.01, ****P < 0.0001. The data are represented as the mean ± SEM

To determine whether the changes in the transcription of synapse-related genes encoding *PSD95*, *Synapsin 1*, *vGLUT1*, and *SNAP25* induced by PFF or A-366 treatment are related to H3K9me2 modification at these gene promoters, we performed ChIP assays using primary neurons. Primers against the proximal and immediate upstream transcription start site regions of genes were designed to measure the occupancy of H3K9 dimethylation at these regions. We found that the enrichment of H3K9 dimethylation at these genes was significantly increased in neurons after PFF treatment, while A-366 treatment attenuated the enrichment of H3K9me2 at the promoters of *SNAP25*, *PSD95*, *vGLUT1* and *Synapsin 1* (Fig. 3A, B). ChIP‒qPCR data also demonstrated significant increases in H3K9me2 enrichment at the promoters of *SNAP25*, *PSD95*, *vGLUT1*, and *Synapsin 1* in neurons after PFF treatment, which was attenuated by treatment with A-366 (Fig. 3C). These data suggest that PFF treatment promotes H3K9 dimethylation at the promoters of several synaptic genes and that A-366 decreases H3K9 dimethylation at the promoters of those genes.

### Inhibition of EHMT1/2 alleviates neuronal death without affecting α-syn oligomerization

**Fig. 4:**
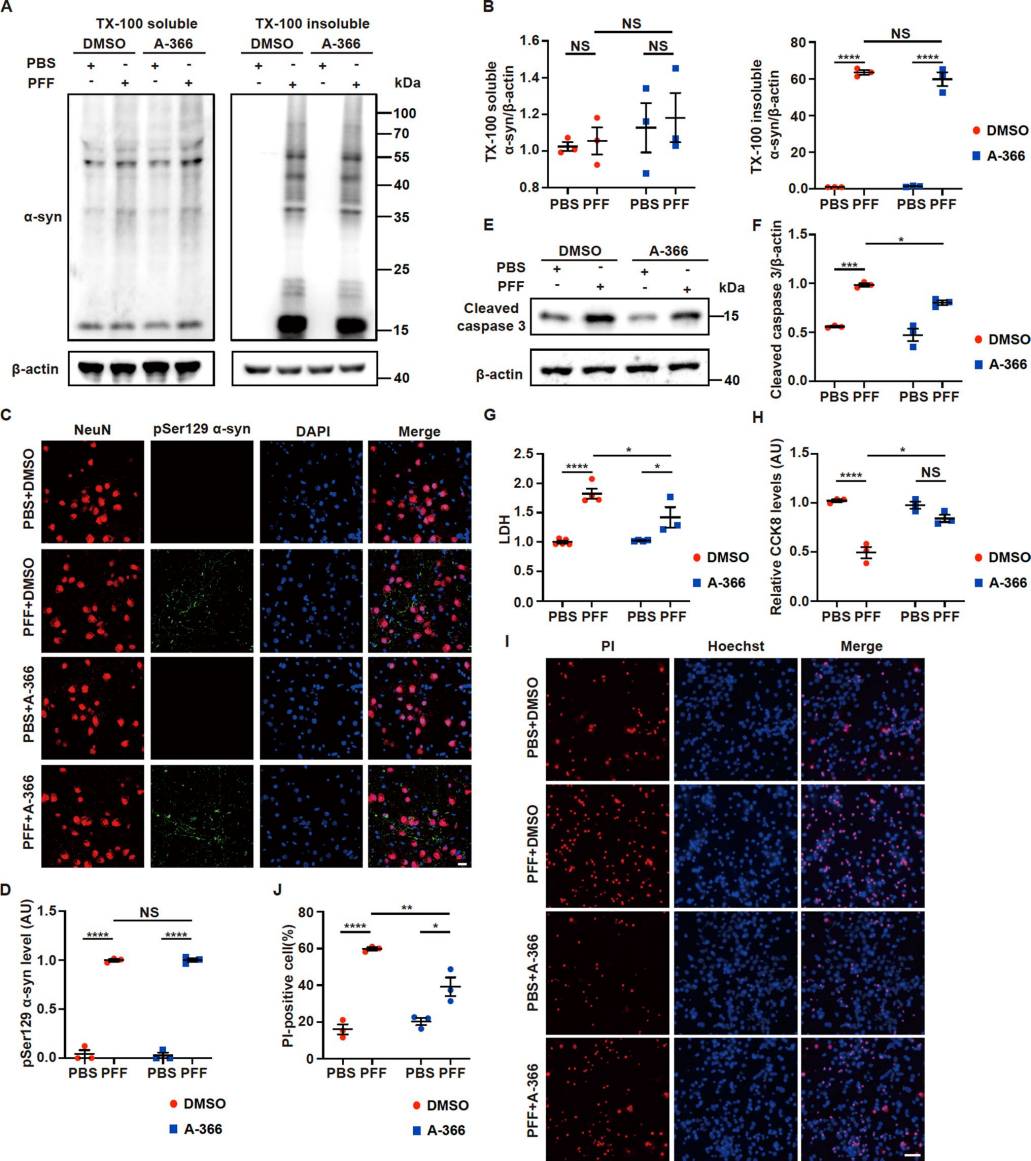
The EHMT1/2 inhibitor A-366 rescues the death of neurons without affecting the formation of α-syn oligomers. Primary neurons were incubated with 3 µg/mL PFFs at 7 DIV for 14 days and then processed for analysis. Treatment with A-366 was administered according to the schematic diagram depicted in Supple Fig. 3C. **A**–**B** Immunoblots and quantitative analysis of Triton X-100-soluble and Triton X-100-insoluble α-syn in primary neurons treated with PFFs, PBS, A-366 or DMSO (n = 3). **C** Representative confocal images of immunofluorescence staining for NeuN and pSer129 α-syn in primary neurons. Scale bar, 20 μm. **D** Plots showing the level of pSer129 α-syn (n = 3). **E**–**F** Immunoblots and quantitative analysis of cleaved caspase 3 in primary neurons treated with PFFs or PBS and A-366 or DMSO (n = 3). **G** Culture media from primary neurons treated with PFFs or PBS and A-366 or DMSO were collected to detect LDH release (n = 3–5). **H** The viability of primary neurons treated with PFFs, PBS, and A-366 or DMSO was measured via the CCK8 assay (n = 3). **I** Representative images of Hoechst and PI staining of primary neurons treated with PFFs or PBS and A-366 or DMSO. Scale bar, 50 μm. **J** Plots showing the percentage of PI-positive cells (n = 3). The *P* values were calculated using two-way ANOVA in **B**, **D**, **F**, **G**, **H** and **J**. *P < 0.05, ***P < 0.001, ****P < 0.0001, NS indicates not significant. The data are represented as the mean ± SEM

We showed that A-366 restored the expression of synaptic proteins in primary neurons that were exposed to PFFs (Fig. 2). We wondered whether A-366 influences α-syn oligomerization. In the HEK293-α-syn-GFP cell line (Fig. S4A, B) or primary neurons (Fig. 4A, B), A-366 treatment did not influence PFF-induced α-syn oligomerization. Similar results were obtained in primary neurons in which EHMT1/2 was knocked down (Fig. S4C, D). In addition, A-366 did not alter the PFF-induced formation of pathogenic α-syn, which was labeled with the pSer129 antibody (Fig. 4C, D).

As we have shown that A-366 restores synaptic protein levels that are impaired by PFFs (Fig. 2), we wondered whether A-366 could reduce neuronal death caused by PFFs in neurons in which α-syn oligomerization still occurs upon PFF treatment. In primary neurons that were treated with PFFs, an increase in caspase 3 cleavage was observed; however, A-366 treatment attenuated PFF-induced caspase 3 cleavage (Fig. 4E, F). Furthermore, the neuronal death induced by PFFs was also alleviated by A-366, as detected by lactate dehydrogenase (LDH) release assays (Fig. 4G), CCK8 assays (Fig. 4H) and PI staining (Fig. 4I, J).

### Treatment with the EHMT1/2 inhibitor A-366 restores synaptic morphology and number of synapses in PFF-treated primary neurons

**Fig. 5:**
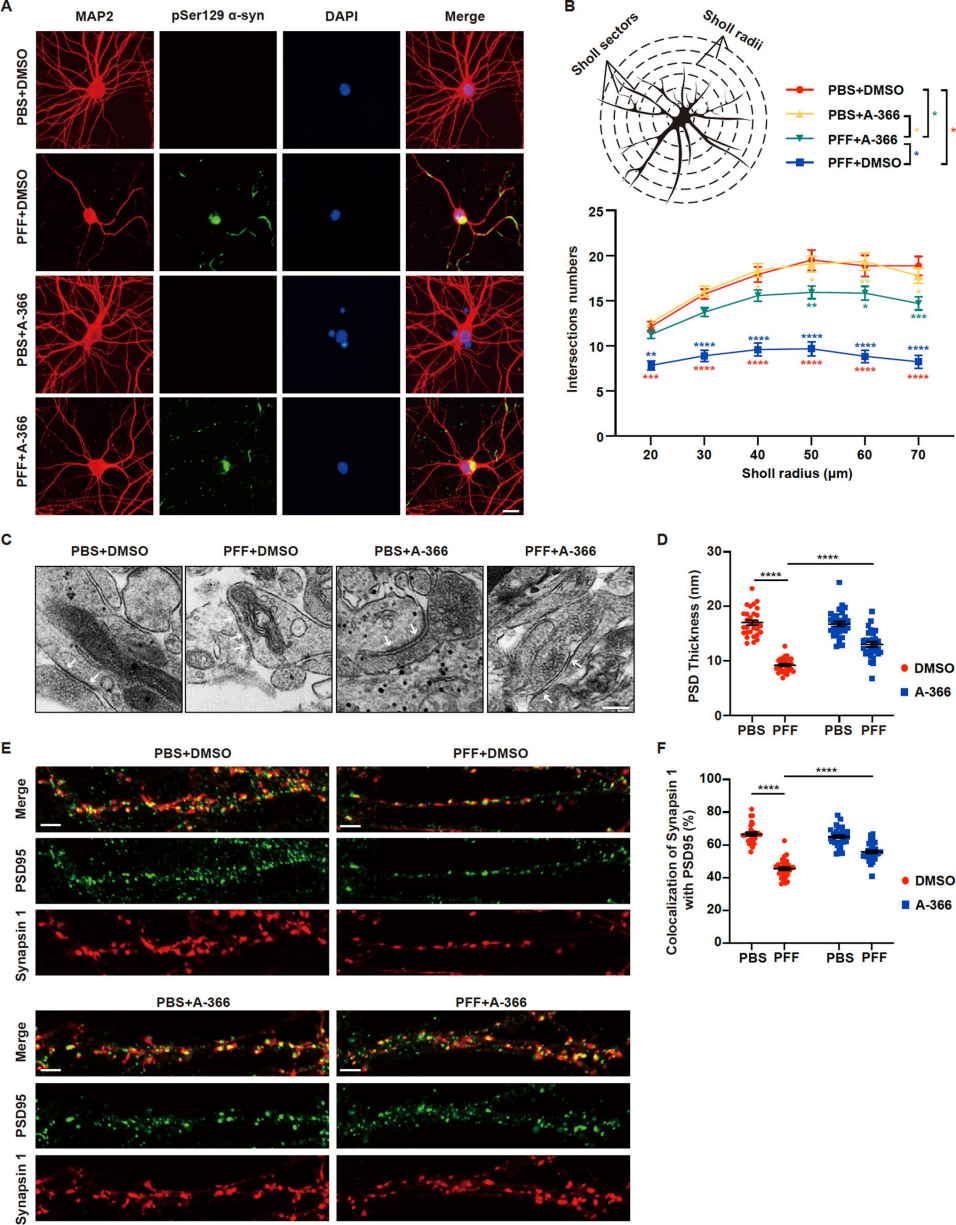
The EHMT1/2 inhibitor A-366 rescues morphological damage and the loss of synaptic numbers in neurons. Primary neurons were incubated with 3 µg/mL PFFs at 7 DIV for 14 days and then processed for analysis. Treatment with A-366 was administered according to the schematic diagram depicted in Supple Fig. 3C. **A** Representative confocal images of immunofluorescence staining for MAP2 and pSer129 α-syn in primary neurons treated with PFFs, PBS, A-366 or DMSO. Scale bar, 20 μm. **B** Diagram of branch intersections in the circular grid and quantification of intersections by Sholl analysis in each group (n = 30). **C** Representative transmission electron microscopy (TEM) images showing the ultrastructure of synapses of primary neurons treated with PFFs or PBS and A-366 or DMSO. Scale bar, 200 nm. **D** Quantification of the thickness of the postsynaptic density (PSD) in each group (n = 30). **E** Representative confocal images of immunofluorescence staining for PSD95 and Synapsin 1 in primary neurons treated with PFFs or PBS and A-366 or DMSO. Scale bar, 5 μm. **F** Quantification of the colocalization of Synapsin 1 with PSD95 in dendritic segments (n = 30). The *P* values were calculated using two-way ANOVA in **B**, **D** and **F**. *P < 0.05, **P < 0.01, ***P < 0.001, ****P < 0.0001. The data are represented as the mean ± SEM

To further determine the protective effects of A-366 on neurons, we examined neurites and synapses from primary neurons that were treated with PFFs alone or in combination with A-366. The morphology of the dendrites and bodies of primary neurons 14 days after exposure to PFFs, was visualized using MAP2 staining (Fig. 5A). We also conducted neuronal Sholl analyses to assess the complexity and morphology of the neurons. As shown in Fig. 5B, the primary neurons treated with PFFs exhibited a significant decrease in the number of intersections, indicating that PFF treatment reduces the branching of neurites (Fig. 5B). However, A-366 treatment significantly restored the number of branches (Fig. 5A, B). In addition, the PFF-induced increase in pathogenic α-syn remained unchanged under A-366 treatment (Fig. 5A), which is consistent with the biochemical data shown in Fig. 4. Ultrastructural characterization of the synaptic compartments via transmission electron microscopy (TEM) revealed intact synapses containing presynaptic vesicles and postsynaptic densities (white arrows) in the PBS or A-366 treatment groups (Fig. 5C). In contrast, decreases in postsynaptic density, increases in dystrophic synaptic structure density and a reduction in thickness of the postsynaptic density were observed in the PFF treatment group of primary neurons (Fig. 5C, D), which is consistent with previous reports that PFFs induce pathologies from axons to dendrites [12]. Furthermore, A-366 significantly restored the synaptic structure and thickness of the postsynaptic bands that were impaired by PFFs (Fig. 5C, D). In addition, we used the presynaptic marker synapsin 1 and the postsynaptic marker PSD95 to label synapses and analyzed the colocalization of these two markers to characterize the number of synapses; the results revealed a significant decrease in the colocalization of synapsin 1 and PSD95 in neurons treated with PFFs compared to those in neurons treated with PBS (Fig. 5E, F). Moreover, the administration of A-366 effectively mitigated the reduction in synaptic number induced by PFF treatment (Fig. 5E, F).

### The EHMT1/2 inhibitor A-366 rescues motor deficits in PFF model mice

**Fig. 6:**
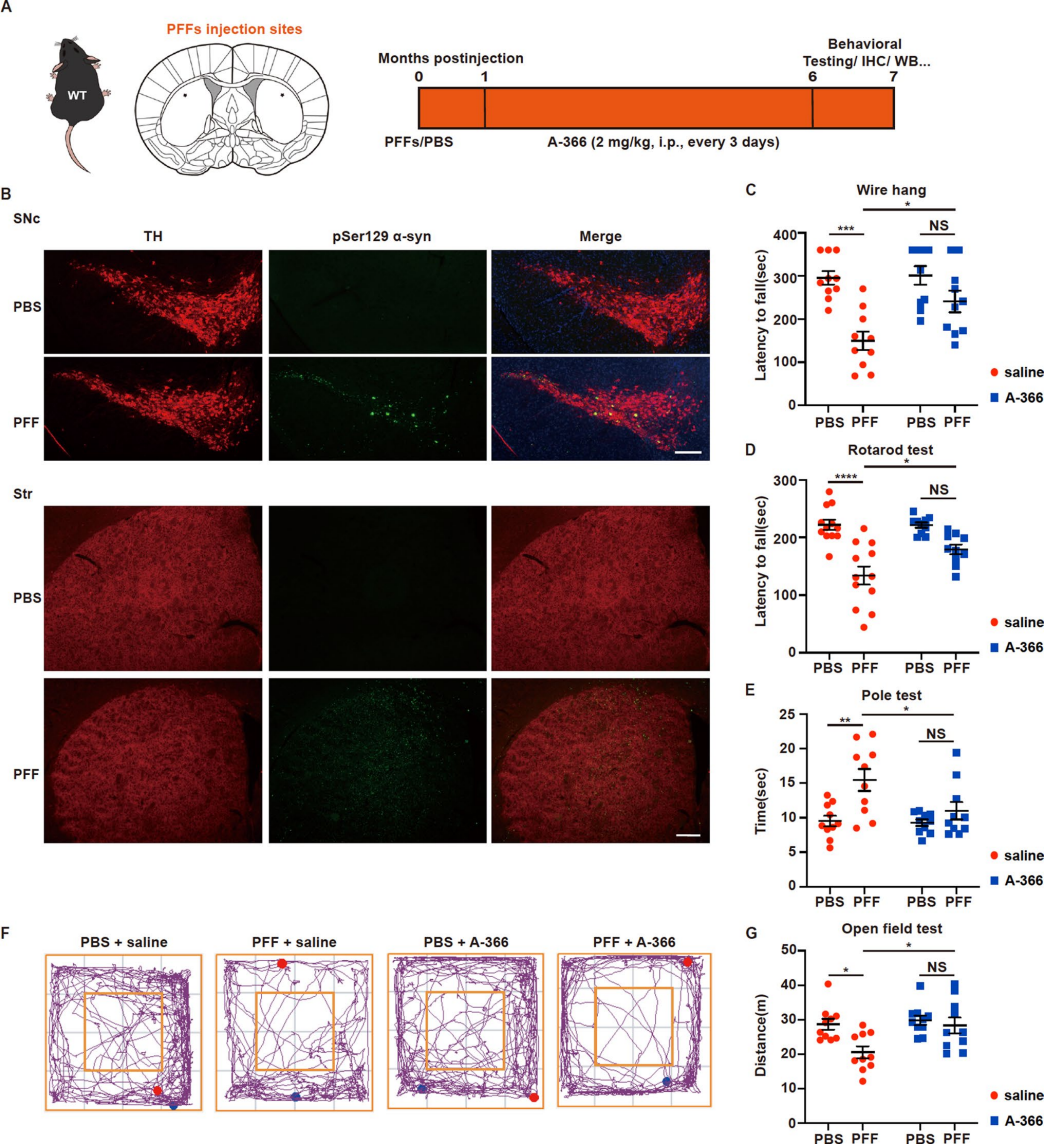
EHMT1/2 inhibition rescues motor deficits in PFF model mice. **A** Scheme the stereotactic injection of PFF and timeline of the A-366 administration. Mice were anesthetized and stereotaxically injected bilaterally into the striatum region with PFFs (2.5 μg/side); PBS was used as a control. **B** Representative images of immunofluorescence staining of TH and pSer129 α-syn within the SNpc and striatum in mice treated with PFFs or PBS. Scale bar, 100 μm. (**C**–**G**) Mice were injected with 5 µg of PFFs. Treatment with A-366 was administered according to the schematic diagram depicted in Fig. 6A. **C**–**E** Behavioral assessment at 6 months after PFF injection. The results for the mice in the **C** wire hang test, **D** rotarod test, and **E** pole test are shown (n = 10–12/group). **F** Representative movement paths of mice from each group in the open field test. **G** The distances traveled by mice treated with PFFs or PBS and A-366 or saline are shown (n = 10/group). The *P* values were calculated using two-way ANOVA in **C**, **D**, **E** and **G**. *P < 0.05, **P < 0.01, ****P < 0.0001. The data are represented as the mean ± SEM

To examine whether inhibition of EHMT1/2 could mitigate the toxicity of PFFs in vivo, we bilaterally and stereotactically injected PFFs into the dorsal striatum of wild-type mice (Fig. 6A). Thirty days after PFF injection, the mice were treated with or without A-366 (2 mg/kg, i.p., every 3 days for 5 months) (Fig. 6A). α-Syn pathology, which was indicated by pSer129 α-syn immunoreactivity, was observed in the striatum and in tyrosine hydroxylase (TH)-positive neurons in the SNpc 30 days after PFF injection (Fig. 6B), suggesting the spread of α-syn pathology from the striatum to the SNpc. Six months after PFF injection, we conducted a series of motor behavioral tests. The mice that received PFF injection exhibited poor performance in the wire hang test, a complementary measure of motor strength and coordination (Fig. 6C), and a nearly 50% decrease in latency compared to that of the control group that received PBS + saline (Fig. 6C). However, the PFF-treated mice that received systemic administration of A-366 exhibited significantly better performance in the wire hang test than did the PFF-treated mice (Fig. 6C). In addition, A-366 alone did not change the performance of the mice compared to that of the control group (Fig. 6C). The rotarod test (Fig. 6D) revealed significant deterioration of performance in PFF mice, indicating an impairment of motor coordination and balance; however, A-366 improved the performance of PFF mice. The pole test (Fig. 6E) and the open field test (Fig. 6F, G) showed decreases in locomotor activity in PFF mice; however, A-366 significantly improved locomotor activity in PFF mice. These motor behavioral tests indicate that systemic administration of A-366 improves motor ability, which is impaired by PFFs.

### The EHMT1/2 inhibitor A-366 decreases the level of H3K9me2 and attenuates the loss of DA neurons in PFF model mice

**Fig. 7:**
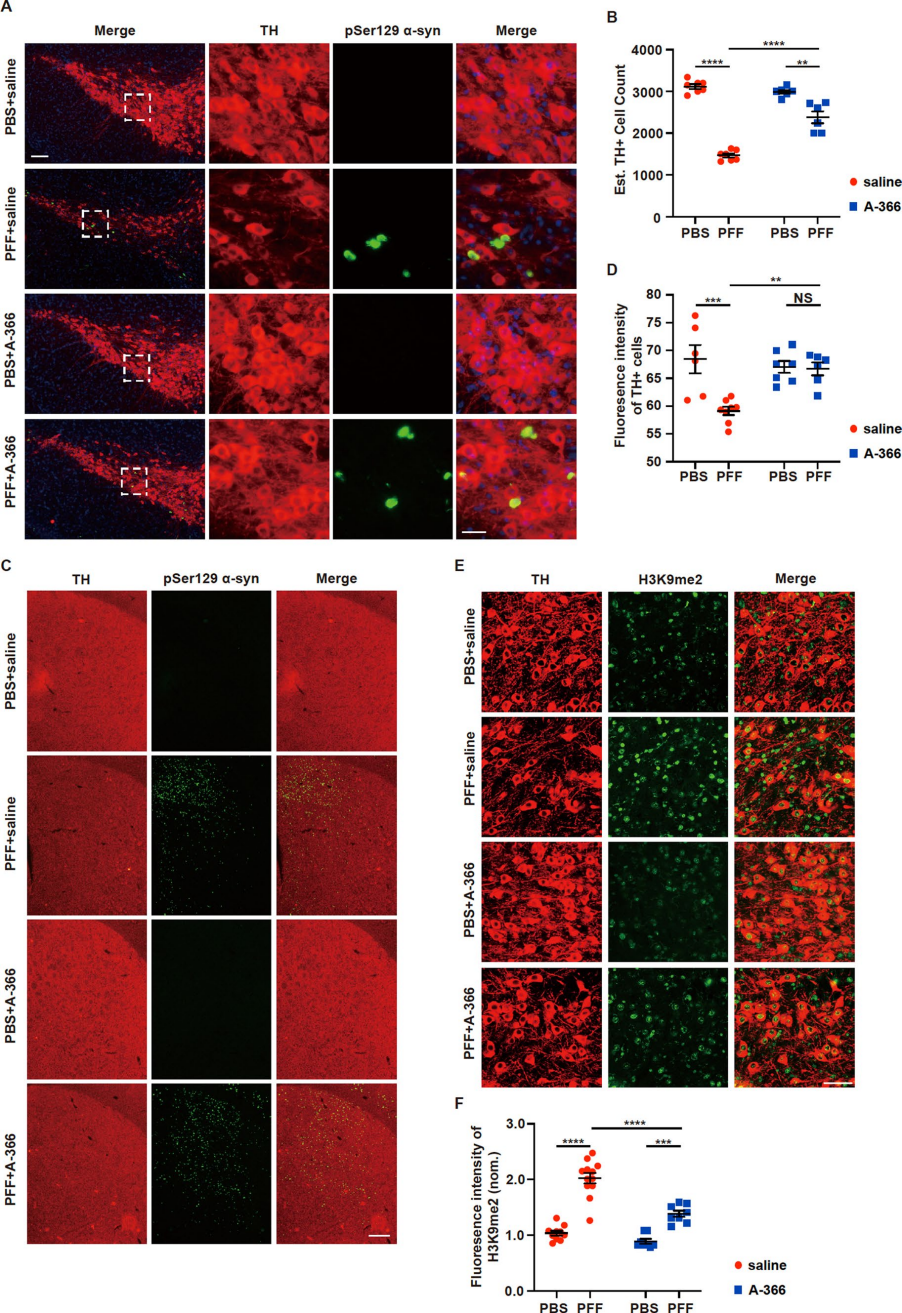
The EHMT1/2 inhibitor A-366 rescues the loss of dopamine neurons by reducing the level of H3K9me2 in PFF model mice. Mice were injected with 5 µg of PFFs. Treatment with A-366 was administered according to the schematic diagram depicted in Fig. 6A. **A** Representative images of immunofluorescence staining of TH and pSer129 α-syn within the SNpc. Scale bar, 100 μm. **B** Quantification of TH-positive neurons within the SNpc (n = 6/group). **C** Representative images of immunofluorescence staining of TH and pSer129 α-syn within the striatum. Scale bar, 25 μm. **D** Quantification of the fluorescence intensity of TH-positive cells in the striatum (n = 6/group). **E** Representative images for immunofluorescence staining of TH and H3K9me2 within the substantia nigra. Scale bar, 50 μm. **F** Quantification of the fluorescence intensity of H3K9me2 in the substantia nigra (n = 8–12/group). The *P* values were calculated using two-way ANOVA in **B**, **D** and **F**. *P < 0.05, **P < 0.01, ***P < 0.001, ****P < 0.0001. The data are represented as the mean ± SEM

To assess α-syn pathology, pSer129 α-syn immunoreactivity in TH-positive neurons was monitored in the SNpc at 6 months after PFF injection (Fig. 6A). A significant loss of DA neurons in PFF mice was observed, and A-366 administration significantly attenuated the loss of DA neurons caused by PFFs (Fig. 7A, B). Similar results were obtained in the striatum of mice that were injected with PFFs and administered A-366, in which TH immunoreactivity was decreased and TH immunoreactivity was restored (Fig. 7C, D). In addition, the administration of A-366 alone did not cause the loss of DA neurons (Fig. 7A–D). We next examined the expression levels of H3K9me2 in mice that were injected with PFFs and treated with or without A-366. Immunohistochemical staining revealed that the intensity of the H3K9me2 signal in TH-positive neurons in the SNpc was significantly increased in PFF mice; however, administration of A-366 significantly reduced the intensity of the H3K9me2 signal (Fig. 7E, F). Furthermore, the abundance of GFAP and IBA1 in the midbrain was increased in PFF mice, and administration of A-366 significantly attenuated the PFF-induced upregulation of GFAP and IBA1 (Fig. S5A, B). Thus, our data indicate that the injection of PFFs into the striatum induces DA-related neuronal death and the upregulation of H3K9me2 in the SNpc of PFF mice and that the administration of A-366 alleviates the upregulation of H3K9me2 and the loss of DA neurons.

### The EHMT1/2 inhibitor A-366 restores synapse-related proteins in PFF model mice

**Fig. 8:**
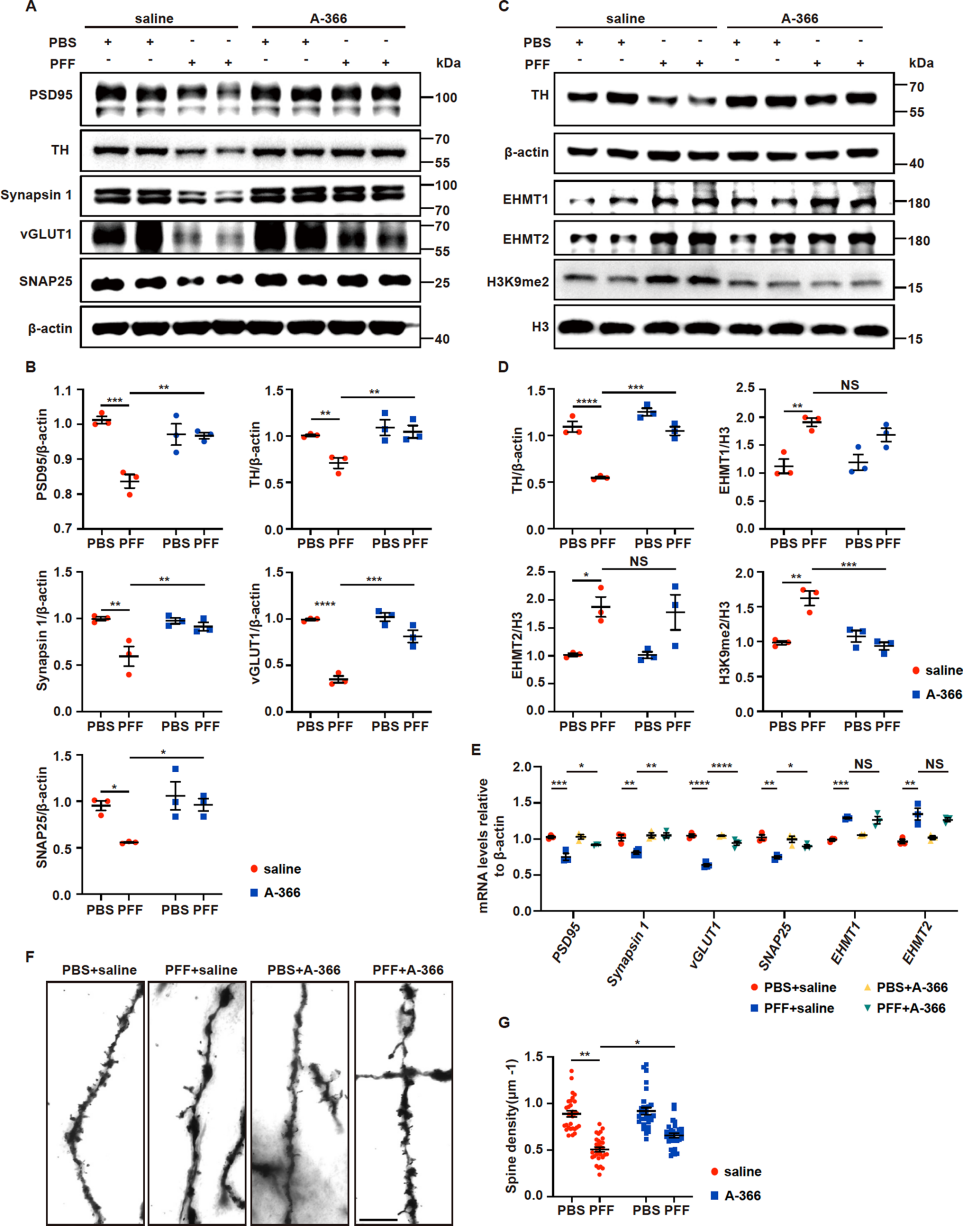
The EHMT1/2 inhibitor A-366 rescues the decrease in synapse-related proteins by reducing the level of H3K9me2 in PFF model mice. Mice were injected with 5 µg of PFFs. Treatment with A-366 was administered according to the schematic diagram depicted in Fig. 6A. **A**–**B** Immunoblots and quantitative analysis of PSD95, Synapsin 1, vGLUT1, SNAP25 and TH in the striatal tissue of mice treated with PFFs, PBS, A-366 or saline (n = 3). **C**–**D** Immunoblots and quantitative analysis of EHMT1, EHMT2, H3K9me2 and TH from midbrain tissue of mice (n = 3). **E** Quantitative real-time RT‒PCR data showing the mRNA levels of *PSD95*, *Synapsin 1*, *vGLUT1*, *SNAP25*, *EHMT1* and *EHMT2* in the midbrain tissue of mice (n = 3). **F** Representative images of Golgi staining of neurons within the substantia nigra in mice treated with PFFs or PBS and A-366 or saline. Scale bar, 10 μm. **G** Quantification of neuronal dendritic spines in the substantia nigra (n = 30/group). The *P* values were calculated using two-way ANOVA in **B**, **D**, **E** and **G**. *P < 0.05, **P < 0.01, ***P < 0.001, ****P < 0.0001, NS indicates not significant. The data are represented as the mean ± SEM

To determine whether the inhibition of EHMT1/2 by A-366 represses the PFF-induced increase in H3K9me2 levels and impacts the expression of synapse-related genes in vivo, we examined gene and protein expression in PFF mice treated with or without A-366. In the striatum, the synapse-related proteins PSD95, Synapsin 1, vGLUT1, and SNAP25 and the DA neuronal marker TH were significantly lower in PFF mice than in control mice; however, A-366 treatment significantly restored the levels of those proteins that were impaired in PFF mice (Fig. 8A, B). In the midbrain, the expression levels of EHMT1/2 and H3K9me2 were increased, and the levels of TH were decreased in PFF mice (Fig. 8C, D). However, A-366 administration significantly reduced H3K9me2 levels and restored the TH levels that were impaired by PFFs but did not alter the increase in EHMT1/2 levels that were induced by PFFs (Fig. 8C, D). We also performed quantitative real-time PCR analyses to examine the mRNA levels of genes that encode synaptic proteins and methyltransferases in the midbrain. In PFF mice, a decrease in the mRNA levels of *PSD95*, *Synapsin 1*, *vGLUT1*, and *SNAP25* and an increase in the mRNA levels of *EHMT1* and *EHMT2* were observed (Fig. 8E). However, A-366 treatment significantly restored the mRNA levels of *PSD95*, *Synapsin 1*, *vGLUT1*, and *SNAP25* but did not alter the levels of *EHMT1* or *EHMT2* in the midbrains of PFF mice (Fig. 8E). To further determine the effects of PFFs on synapses in vivo, we performed Golgi staining to detect synapses in the SN. The number of dendritic spines was significantly reduced upon PFF injection, but A-366 significantly restored the spine number in PFF mice (Fig. 8F, G). In addition, the number of synapses was decreased in the striatum of PFF mice, as indicated by immunostaining with antibodies against PSD95 and synapsin 1 (Fig. S6A, B). However, A-366 significantly restored the number of synapses that was impaired in PFF mice (Fig. S6A, B). These data indicate that inhibition of EHMT1/2 by A-366 protects neurons against PFF-induced loss of synapses.

## Discussion

Despite the unclear etiology of PD, a combination of genetic risk factors and environmental factors may underlie PD pathogenesis [41–43]. Many genes are involved in the disease process, and the deregulation of those genes might be mediated by epigenetic regulation [44–48]. Evidence from PD brains and blood samples suggests alterations in DNA methylation during progression of disease [49]. Epigenetic regulation is involved in many cellular processes, including the regulation of autophagy, the modulation of neuroinflammation, the misfolding of proteins, and the activities of mitochondria and lysosomes, which are associated with the onset of PD [23, 50–52]. Although many studies indicate alterations in DNA methylation and histone acetylation in PD, the regulation of histone methylation has rarely been reported [21, 23, 24, 48, 53–57]. The present study demonstrated that PFF-induced α-syn pathology promotes H3K9 dimethylation by increasing the expression of the methyltransferase EHMT1/2 in primary neurons and PD model mice, leading to decreases in the expression of synaptic proteins and impairments in synapses.

Histone methylation plays a key role in maintaining transcriptional homeostasis via the activation and repression of gene transcription [58, 59]. H3K9me2 is a marker of gene transcriptional repression that is catalyzed by EHMT1 and EHMT2. The functions of EHMT1 and EHMT2 have been implicated in neurodevelopmental disorders, including Kleefstra syndrome and Prader-Willi syndrome, and EHMT1 has been identified as a schizophrenia susceptibility gene [35, 60–62]. The EHMT1/2-mediated H3K9me2 modification is involved in brain functions associated with synaptic networks, including dendritic spine plasticity, locomotor behavior, learning and memory consolidation [26, 63–65]. *GluA2* and *NR2B* (glutamate receptor subunits), *Pcdhb14* and *Pcdhb15* (regulators of neuronal diversity), *Arc* (a prominent factor of synaptic plasticity) and *SNAP25* (a component of the SNARE complex) are regulated by EHMT1/2-mediated H3K9me2 modification [33, 37, 39, 66]. These studies suggest the involvement of histone methylation by EHMT1/2 in synaptic functions.

Defects in synaptogenesis and dysfunction in neuronal communication are common features of neurological diseases [56]. In PD, synaptic dysfunction can be induced by α-syn. It has been reported that α-syn overexpression or aggregation leads to the loss of synapses [67]. To date, the regulation of histone methylation by α-syn has rarely been reported. The only report on this topic is that overexpression of α-syn in SH-SY5Y cells induces an increase in H3K9 dimethylation, leading to downregulation of SNAP25 [39]. Moreover, inhibition of EHMT2 decreases the α-syn-induced increase in H3K9me2 and restores SNAP25 protein levels [39]. In addition, in a study in which trans-ethnic fine-mapping of the major histocompatibility complex region was used for European populations (14,650 cases and 1,288,625 controls) and East Asian populations (7712 cases and 27,372 controls), *EHMT2* was found to be related to PD [38]. In our study, we found that PFFs upregulate H3K9me2 and EHMT1/2 in both primary neurons and PFF mice. In addition to SNAP25, other synaptic proteins, such as PSD95, Synapsin 1 and vGLUT1, are downregulated in PFF-treated primary neurons and in PFF mice. In our study, we used A-366 to specifically inhibit EHMT1/2, which has significantly less cytotoxic effects than does the inhibitor UNC0638 [40, 68]. A-366 has been reported to exhibit improved drug inhibitory effects compared to UNC0642 in animal models [69]. We found that synaptic defects and protein expression were attenuated by A-366 in PFF-treated neurons and mice. Interestingly, the formation and spread of pathological α-syn were not altered by the inhibition of EHMT1/2 with A-366. Moreover, A-366 improved the motor activities that were impaired by PFFs. We also observed that A-366 decreased the enrichment of H3K9me2 at the promoters of several synaptic genes, including *PSD95*, *Synapsin 1*, *SNAP25* and *vGLUT1*. Thus, our data suggest that the effects of epigenetic alterations on synapses in neurons and mice occur downstream of PFF pathology.

Mitochondrial dysfunction at the synaptic site may be a critical factor contributing to synaptic injury in PD patients [70, 71]. Defective mitochondrial ATP generation is a primary factor leading to early synaptic energy depletion and is associated with synaptic dysfunction [72]. Furthermore, mitochondrial dysfunction is closely linked to the activation of caspase-3 and neuronal death. We observed that A-366 restored that expression levels of synaptic proteins, which were reduced by PFF treatment, and attenuated PFF-induced neuronal death in vitro. As EHMT1 also regulates the expression of mitochondrial-related genes, as well as oxygen consumption and ATP production [73], the protective effects of A-366 on PFF-treated neurons may be due to not only the epigenetic regulation of synaptic genes but also mitochondrial gene expression though the repression of EHMTs by A-366. Further studies on the role of EHMT1 in PFF-mediated mitochondrial dysfunction may elucidate the regulatory mechanisms involved synaptic regulation.

**Fig. 9:**
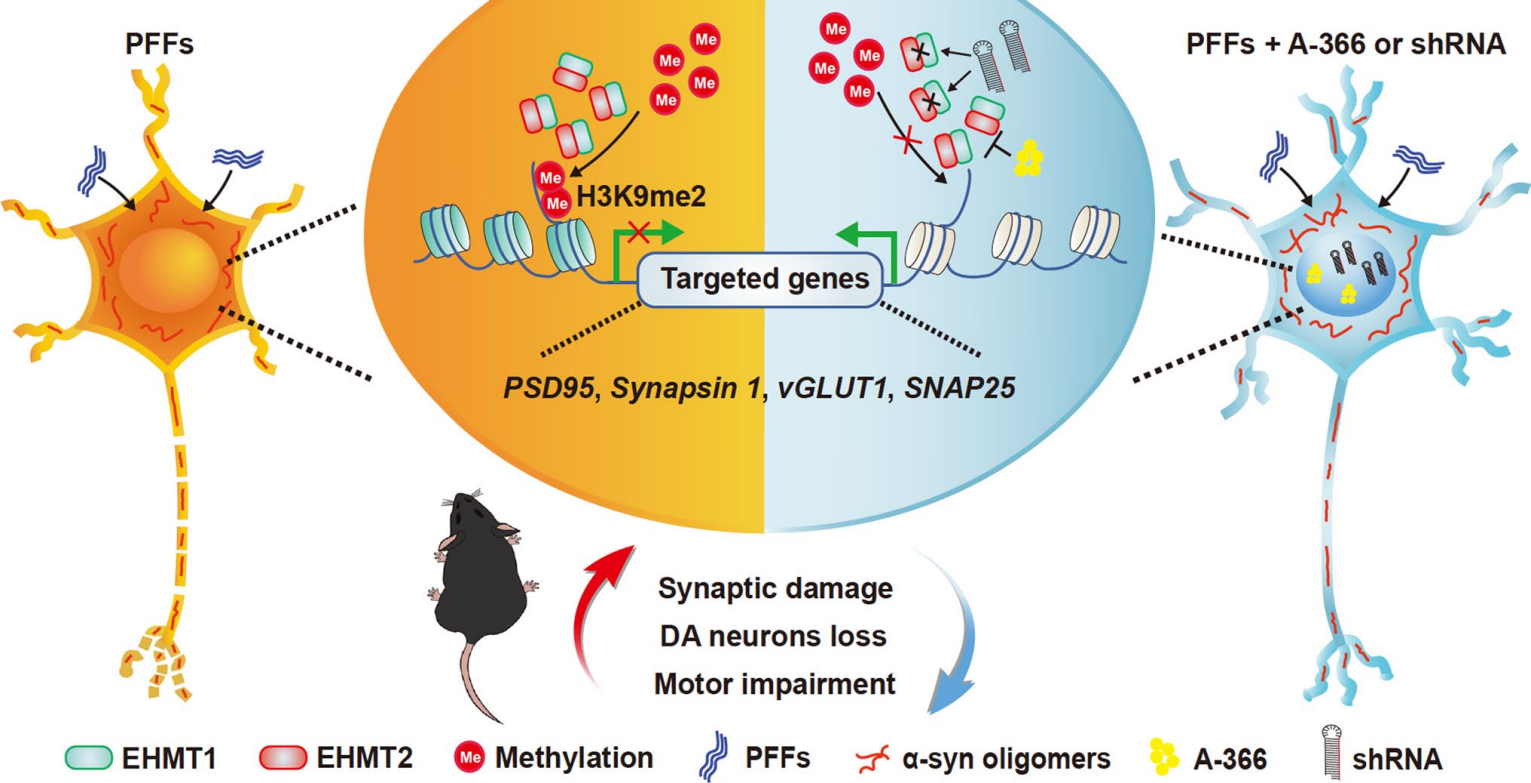
A schematic model showing the epigenetic mechanism of PD. PFFs induce the accumulation of α-syn oligomers and lead to the increased H3K9me2 repressive histone methylation at the promoter regions of *SNAP25*, *PSD95*, *Synapsin 1*, and *vGLUT1*, causing decreased transcription of these genes (left side). Consequently, synaptic damage, DA neuron loss and motor impairment occur in PD mice. Inhibiting EHMT1/2 with the inhibitor A-366 or shRNA reversed the aberrant epigenetic regulation of synapses in PD models, restoring synaptic function and motility (right side)

In summary, our study provides a mechanistic link between α-syn pathology and histone methylation, which induces defects in the expression of synaptic proteins and impairments in locomotion. Inhibition of the histone methyltransferases EHMT1 and EHMT2 restored synaptic protein levels and improved animal behaviors that were impaired by PFFs but did not influence the formation or propagation of α-syn pathology (Fig. 9). Thus, our study provides evidence that H3K9me2 modification by EHMT1 and EHMT2 is involved in synaptic damage associated association with α-syn pathology and indicates that the effects of epigenetic alterations on synapses in neurons and mice are downstream of PFF pathology.

## Materials and methods

### Recombinant α-syn purification and in vitro fibrillization

Full-length α-syn with an N-terminal His-tag was expressed in BL21 cells and purified according to purification methods described elsewhere [74]. The recombinant α-syn monomer was verified using Coomassie blue staining. Fibrillization of α-syn was prepared using recombinant α-syn at a concentration of 3 mg/mL in sterile Dulbecco’s PBS and incubated at 37 °C with constant agitation at 1000 rpm for 7 days. The fibrillization of α-syn was verified via electron microscopy. The seed properties of PFFs were verified by Western blotting and immunocytochemistry in primary neurons. Assembled PFFs were aliquoted and stored at −80 °C.

### Primary neuronal cultures

Primary neuronal cultures were prepared from E16–E18 C57BL/6J mouse brains. All procedures were performed according to the institutional guidelines for the use and care of animals and were approved by the ethical committee of Soochow University. Dissociated cortical neurons were plated onto poly-D-lysine-coated coverslips or dishes at 30,000–60,000 cells/cm^2^.

### α-Synuclein fibril transduction and drug treatment or lentivirus treatment

PFFs were diluted in PBS and sonicated before addition. Owing to the lack of receptors located on the neuronal membrane for the active uptake of PFFs by HEK293 cells, we transfected PFFs into HEK293-α-syn-GFP cells at a concentration of 3 µg/mL with Lipofectamine 3000 reagents at a density of 30% – 40% and treated them for 72 h. Primary neurons were incubated with 3 µg/mL PFFs for 14 days. The inhibitor A-366 purchased from Selleck (Catalog no. S7572) was added at a dose of 2 µM after PFF transfection for 24 h to treat HEK293-α-syn-GFP cells for 48 h. In primary neurons, A-366 was added at a dose of 2 µM at 9 DIV after PFF transduction for 48 h and was added every three days for 12 days for cell death and viability assessment. Short hairpin RNA oligonucleotides targeting the mouse *Ehmt1 (1)* sequence (*5′-*TTATTCATCATCATCATAGCG-*3′*), *Ehmt1 (2)* sequence (*5′-*CGCTATGATGATGATGAATAA-*3′*) or *Ehmt2 (1)* sequence (*5′-*AAGAGCTATGAACTCTCTCGG-*3′*), *Ehmt2 (2)* sequence (*5′-*CCGAGAGAGTTCATAGCTCTT-*3′*) were added together to primary neurons at 5 DIV, and scr. shRNA was used as a control. Then, PFFs were added to primary neurons at 7 DIV for 14 days.

### Nuclear fragmentation and western blotting

The cells were collected and then homogenized with 200 μL of homogenization buffer (20 mM Tris–HCl, pH 7.4; 10 mM NaCl; 3 mM MgCl_2_; 0.5% NP-40; 1 mM PMSF; and cocktail protease inhibitor). Mouse brain slices were homogenized with 500 μL of homogenization buffer. The homogenates were incubated on ice for 15 min, followed by centrifugation at 3000 × g at 4 °C for 10 min. The nuclear pellets were resuspended in 50 μL of nuclear extraction buffer (100 mM Tris–HCl, pH 7.4; 100 mM NaCl; 1 mM EDTA; 1% Triton X-100; 0.1% SDS; 10% glycerol; 1 mM PMSF; and cocktail protease inhibitor) and incubated on ice for 30 min with periodic vortexing to resuspend the pellets. After centrifugation, the supernatant was collected, boiled in 2 × loading buffer for 10 min and then separated on 8–12% SDS‒polyacrylamide gels. Western blotting experiments for nuclear proteins was performed with antibodies against EHMT1 (1:1000; NOVUS, NBP1-77400SS), EHMT2 (1:1000; Abcam, ab185050), H3K9me2 (1:1000; Abcam, ab1220), H3K9me3 (1:3000; Abcam, ab176916), H3K4me3 (1:1000; Abcam, ab213224) and H3 (1:1000; Beyotime, AF0009).

Total proteins from cells and mouse brains were homogenized and sonicated with cell lysis buffer (1 M Tris–HCl, pH 7.6; 5 M NaCl; 10% NP40; 5% sodium deoxycholate; and cocktail protease inhibitor) and then incubated on ice for 15 min, followed by centrifugation. After centrifugation, the supernatant was collected, boiled in 2 × loading buffer for 10 min and then separated on 8–12% SDS‒polyacrylamide gels. Western blotting of total proteins was performed using antibodies against α-synuclein (1:1000; BD Biosciences, 610787), β-actin (1:10,000; Sigma, A1978), cleaved caspase 3 (1:500; CST, 9661S), GFP (1:3000; Santa Cruz, sc-9996), PSD95 (1:1000; CST, 3450T), synapsin 1 (1:1000; Synaptic Systems, 106011), SNAP25 (1:1000; Santa Cruz, sc-20038), TH (1:1000; Proteintech, 25859-1-AP), and vGLUT1 (1:1000; Millipore, MAB5502).

### Sequential extraction and Western blot analyses

The neurons were scraped into 1% Triton X-100 in Tris-buffered saline (TBS) (50 mM Tris, 150 mM NaCl, pH 7.4) supplemented with protease and phosphatase inhibitor cocktail at 4 °C. The samples were sonicated 10 times at a 0.5 s pulse and at 100 W power and incubated on ice for 30 min. After centrifugation at 16,000 g and 4 °C for 30 min, the supernatant was collected as the Triton X-100 soluble fraction. The precipitate was resuspended in 1% Triton X-100 buffer and centrifuged once more, after which the pellet was collected as the Triton X-100 insoluble fraction. The pellets were resuspended in 2% SDS in Tris-buffered saline (TBS) supplemented with protease and phosphatase inhibitor cocktail lysis buffer, sonicated 15 times at a 0.5 s pulse and at 100 W power and centrifuged at 20,000 × g for 30 min. The samples were boiled in 4 × loading buffer for 10 min and then separated on 12% SDS‒polyacrylamide gels. Western blotting was performed with antibodies against α-synuclein (1:1000; BD Biosciences, 610787) and β-actin (1:10,000; Sigma, A1978).

### Quantitative real-time PCR

Total RNA was isolated from primary neurons and mouse brain slices using TRIzol reagent (Invitrogen). Then, cDNA was subsequently synthesized using the PrimeScript™ RT reagent Kit (Takara). Quantitative PCR was carried out using SYBR™ Green PCR Master Mix (Invitrogen) according to the manufacturer’s instructions. In brief, actin was used as the housekeeping gene for quantitation of the expression of target genes in neurons and mice. Fold changes in the expression of the target genes were determining the fold change = 2−Δ(ΔCT), where Δ CT − CT (target) − CT (actin), and Δ (Δ CT) − Δ CT (treated group) −Δ CT (PBS + DMSO). CT (threshold cycle) was defined as the fractional cycle number at which the fluorescence reached 10 × the s.d. of the baseline. A mixture of 20 µl of PCR per well was amplified according to the following cycling procedure: 95 °C for 5 min, followed by 40 cycles of 95 °C for 30 s, 55 °C for 30 s and 72 °C for 60 s. The sequences of the primers are used summarized in the Supplementary Table 1.

### Quantitative chromatin immunoprecipitation assay

Chromatin immunoprecipitation products were prepared according to the manufacturer’s instructions (SimpleChIP® Enzymatic Chromatin IP Kit, CST, 9003) with modifications. Briefly, nuclear proteins were cross-linked to DNA by adding formaldehyde directly to the medium to a final concentration of 1% (v/v) for 10 min. Cross-linking was stopped by adding glycine to a final concentration of 0.125 M and incubating at room temperature (RT) for 5 min. The medium was removed, and the cells were washed twice with cold PBS. The cells were collected by scraping into cold PBS supplemented with a protease inhibitor cocktail. Then, SDS lysis buffer containing proteinase inhibitors was used to homogenize the cells, followed by DNA digestion. Chromatin fragments (150–900 bp in length) were obtained by sonication at 2% power with 2 rounds of 20 s pulses (30 s pause between pulses), after which the samples were immersed in an ice–water bath. After centrifugation at 9400 × g for 10 min at 4 °C, the sheared chromatin was collected and incubated with an antibody against H3K9me2 (1:100; Abcam, ab1220) overnight at 4 °C with gentle rotation, and mouse IgG was used as the negative control. Immunocomplexes were precipitated by ChIP-Grade Protein G Magnetic Beads, followed by sequential washes with low-salt, high-salt, LiCl, and Tris–EDTA buffer. The protein‒DNA crosslinking was reversed by incubation with 5 M NaCl and proteinase K at 65 °C for 4 h, after which the DNA fragments were purified using DNA purification columns.

Both regular and real-time PCR were performed to amplify 100–200 bp fragments around the transcription start site region of mouse *PSD95*, *Synapsin 1*, *vGLUT1* and *SNAP25*. The primer sets used were as follows: *PSD95: 5′*-TGAGATCAGTCATAGCAGCTACT-*3′*; *5′*- CTTCCTCCCCTAGCAGGTCC-*3′*; *Synapsin 1:5′*-AGCTCAACAAATCCCAGTCTCT-*3′*; *5′*-CGGATGGTCTCAGCTTTCAC-*3′*; *vGLUT1: 5′*-TTGTGGCTACCTCCACCCTAA-*3′*; *5′*-CAGCCGACTCCGTTCTAAGG-*3′*; *SNAP25: 5′*-ATCCGCAGGGTAACAAATGATG-*3′*; *5′*-CGGAGGTTTCCGATGATGC-*3′*. Quantification of ChIP signals was calculated as a percent of the total input. The standard PCR cycling conditions were as follows: 95 °C for 5 min; 35 cycles of 95 °C for 30 s, 52–60 °C (according to the primers) for 30 s and 72 °C for 13–30 s (according to the primers); and a final extension at 72 °C for 10 min. The ChIP‒qPCR values were normalized to those of the input control and represented as fold enrichment relative to the anti-normal mouse IgG control.

### Behavioral tests

Mice were tested on a comprehensive behavioral test battery 2 weeks prior to sacrifice. The experimenter was blinded to the treatment group for all behavioral studies. All tests were performed and recorded between 10:00 and 16:00 in the lights-on cycle. Mice were habituated to the testing room 1 h before the tests, and the apparatus was cleaned with 30% ethanol between animals to minimize odor cues.

### Wire hang test

The mice were placed on top of a standard wire cage lid. The lid was lightly shaken to cause the animals to grip the wires, after which the wires were turned upside down. The latency of the mice to fall off the wire grid was also measured. Trials were stopped if the mouse remained on the lid after 10 min.

### Rotarod test

For the rotarod test, the mice were trained 1 day before the test. The next day, the mice were placed on an accelerating rotarod cylinder, and the latency of the animals was measured. The speed was slowly increased from 4 to 40 rpm within 5 min. Each mouse was subjected to three consecutive trials, and the mean latency to fall was used in the analysis.

### Pole test

The pole was composed of a 75 cm long wooden rod with a diameter of 9 mm and a 2.5 cm diameter ball at the top of the rod. The wooden rod was wrapped with bandage gauze. The mice were trained for two consecutive days before the actual test. On the test day, the mice were placed on the top of the pole, and the time from the top to the bottom of the rod was recorded. Each mouse was subjected to three consecutive trials, and the mean latency to fall was used in the analysis.

### Open field test

The field was subdivided into peripheral and central sectors, where the central sector included 4 central squares and the peripheral sector included the remaining squares. During the test, the environment was kept quiet, the mice were put into the open field, and their movement was recorded within 15 min using the monitoring software system to obtain the movement-related data. The apparatus was thoroughly cleaned with diluted 30% ethanol between each trial.

### Immunocytochemistry

The neurons were fixed with 4% paraformaldehyde (PFA) for 10 min at RT and washed three times in PBS. The cells were permeabilized in 3% fetal bovine serum supplement with 0.3% Triton X-100 in PBS for 12 min at RT. After a PBS wash, the cells were blocked for 60 min with 3% fetal bovine serum in PBS prior to incubation with primary antibodies overnight at 4 °C. The primary antibodies used were as follows: Ser129-phosphorylated α-synuclein (1:1000; Abcam, ab51253), H3K9me2 (1:300; Abcam, ab1220), MAP2 (1:500; Millipore, MAB3418), NeuN (1:500; Millipore, MAB377), p62 (1:500; Sigma–Aldrich, P0067), PSD95 (1:300; CST, 3450T) and Synapsin 1 (1:1000; Synaptic Systems, 106011). After incubation, the cells were washed 3 times with PBS and incubated with secondary antibodies for 1 h at RT. After a 3 washes with PBS, the cells were incubated with DAPI (1:10,000; Sigma‒Aldrich, D9542) in PBS. Images were captured on an AIR HD25 laser confocal microscope (Nikon) and then processed with Photoshop software (Adobe) and Fiji software (National Institutes of Health).

### Immunohistochemistry

After perfusion and fixation, the brains were embedded in OCT agents and cut into 20 µm sections. Sections were then stained using standard immunohistochemistry as described below. The sections were permeabilized in 0.4% Triton X-100 + 1% fetal bovine serum in PBS for 20 min at RT. The sections were incubated in blocking solution (1 × PBS with 0.4% Triton X-100 and 5% fetal bovine serum) for 1 h. The sections were then incubated with the following primary antibodies in permeable buffer: Ser129-phosphorylated α-synuclein (1:1000; Abcam, ab51253), GFAP (1:1000; Millipore, MAB3402C3), H3K9me2 (1:100; Abcam, ab1220), IBA1 (1:1000; Wako Chemicals, 019-19742), PSD95 (1:200; Abcam, ab12093), Synapsin 1 (1:200; Synaptic Systems, 106011), TH (1:1000; CST, 45648S), and TH (1:1000; Proteintech, 25859-1-AP) overnight at 4 °C. After washing, the sections were incubated with secondary antibodies for 1 h at RT. After 3 washes with PBS, the sections were incubated with DAPI (1:10,000; Sigma‒Aldrich, D9542) in PBS for 10 min. Finally, the sections were mounted with antifade reagent (Beyotime Biotechnology) on glass slides. The sections were imaged using a microscope (IX71, Olympus) or a laser confocal microscope (AIR HD25, Nikon) and then analyzed with Photoshop software (Adobe) and Fiji software (National Institutes of Health). Quantitative analysis of TH + cells was conducted following the methods described elsewhere [75]. Every 10th slide through the substantia nigra pars compacta (SNpc) was stained with tyrosine hydroxylase (TH). The sum of all sections was multiplied by 10 to estimate the total count that would be obtained by counting each section.

### Cell viability assessment

The percentage of cell death was determined by staining with Hoechst 33342 dye (Sigma Aldrich, B2261) and propidium iodide (PI) (Beyotime Biotechnology, ST511) at a dose of 1 µg/mL in neuronal culture medium for 10 min. Images were taken with a fluorescence microscope (ECLIPSE Ti2, Nikon) and then processed and counted with Photoshop software (Adobe) and Fiji software (National Institutes of Health). Cytotoxicity was measured via LDH release analyses using a CytoTox 96 Non-Radioactive Cytotoxicity Assay (Promega, Madison, WI, United States). Briefly, 50 µL of growth medium was mixed with 50 µL of CellTiter-Glo and shaken for 20 min at RT. Then, luminescence was measured to evaluate cell cytotoxicity. Cell viability was determined with a Cell Counting Kit-8 (APExBIO Technology LLC, Houston, USA) according to the manufacturer’s instructions. Briefly, the cells were incubated with 10% CCK8 reagent at 37 °C for 3 h. The absorbance was detected at 450 nm by a microplate reader to determine cell viability.

### Transmission electron microscopy analysis

TEM analysis of PFFs. Briefly, 100 μL of PFF solution was deposited on Formvar-coated 200 mesh copper grids. After two washes with double-distilled H_2_O, the grids were stained with phosphotungstic acid followed by vacuum drying from the edge of the grids. Specimens were examined using TEM.

TEM analysis of neuronal synapses. Briefly, neurons were washed with cold PBS and then fixed with 2.5% glutaraldehyde for 30 min at RT. The neurons were collected and subjected to subsequent sample processing and image acquisition. PSD thickness was analyzed using Fiji software.

### Golgi staining

The dendritic spine density of the mice was determined using Golgi staining with the FD Rapid GolgiStainTM Kit (FD NeuroTechnologies, PK401) according to the manufacturer’s instructions. The sections were imaged on a laser confocal microscope (AIR HD25, Nikon). Confocal z-stack images were taken every 30 s during recording. Images were z-stacked, projected and analyzed with Fiji software (National Institutes of Health).

### Experimental animals

Male C57BL/6J mice (2 months) were purchased from Shanghai Jihui Laboratory Animal Care Company. Mice were group-housed with ad libitum access to food on a 12-h light–dark cycle (light: 6 am–6 pm; dark: 6 pm –6 am). Mice were anesthetized and stereotaxically injected bilaterally into the striatum region with PFFs (5 μg), which were diluted in sterile PBS and sonicated briefly before intracerebral injection. Mice that received PBS injection were used as controls. Injections were performed using a 10 μL syringe (Hamilton, NV) at a rate of 0.1 μL per min with the needle in place for 8 min at each target. The striatum injection site coordinates were as follows: + 0.2 mm relative to bregma,  ± 2.0 mm from midline, and 2.6 mm beneath the dura. One month after PFF injection, all animals were randomly assigned to 4 groups for drug treatment: (1) the PBS + saline group, (2) the PFF + saline group, (3) the PBS + A-366 group and (4) the PFF + A-366 group. Mice in groups (3) and (4) were administered A-366 (2 mg/kg, every 3 days) via intraperitoneal injection for 5 months. Mice in groups (1) and (2) were administered an equal volume of saline. All animal experiments were carried out according to the institutional guidelines for the use and care of animals, and all procedures were approved by the ethical committee of Soochow University.

### Statistical analysis

Fiji software (National Institutes of Health) was used for quantification of the data, and GraphPad Prism 8.00 (GraphPad Software, Version X; La Jolla, CA, USA) was used for statistical analysis and graphing. Significant differences were evaluated using a two-tailed unpaired t test, one-way ANOVA or two-way ANOVA followed by Tukey’s multiple comparisons test. The criterion of significance was set at *P* < 0.05. The values are shown as the mean ± SEM.

### Supplementary Information

Below is the link to the electronic supplementary material.

Supplementary file1 (DOCX 5509 KB)

## Data Availability

The authors declare that all the original data related to the figures and supplementary materials published in this article are available upon rationale request to the corresponding author. All related data are included in either the manuscript or supplementary information.
