# Supplementary material for: Inhibition of EHMT1/2 rescues synaptic damage and motor impairment in a PD mouse model
Source: Cell Mol Life Sci. 2024 Mar 12;81(1):128. doi: 10.1007/s00018-024-05176-5 (PMC10933175; doi:10.1007/s00018-024-05176-5)
Supplement: Supplementary file 1 — Supplementary file1 (DOCX 5509 KB) [file 18_2024_5176_MOESM1_ESM.docx]

**SUPPLEMENTARY FIGURES AND FIGURE LEGENDS**

**Supplementary Figure 1**

**
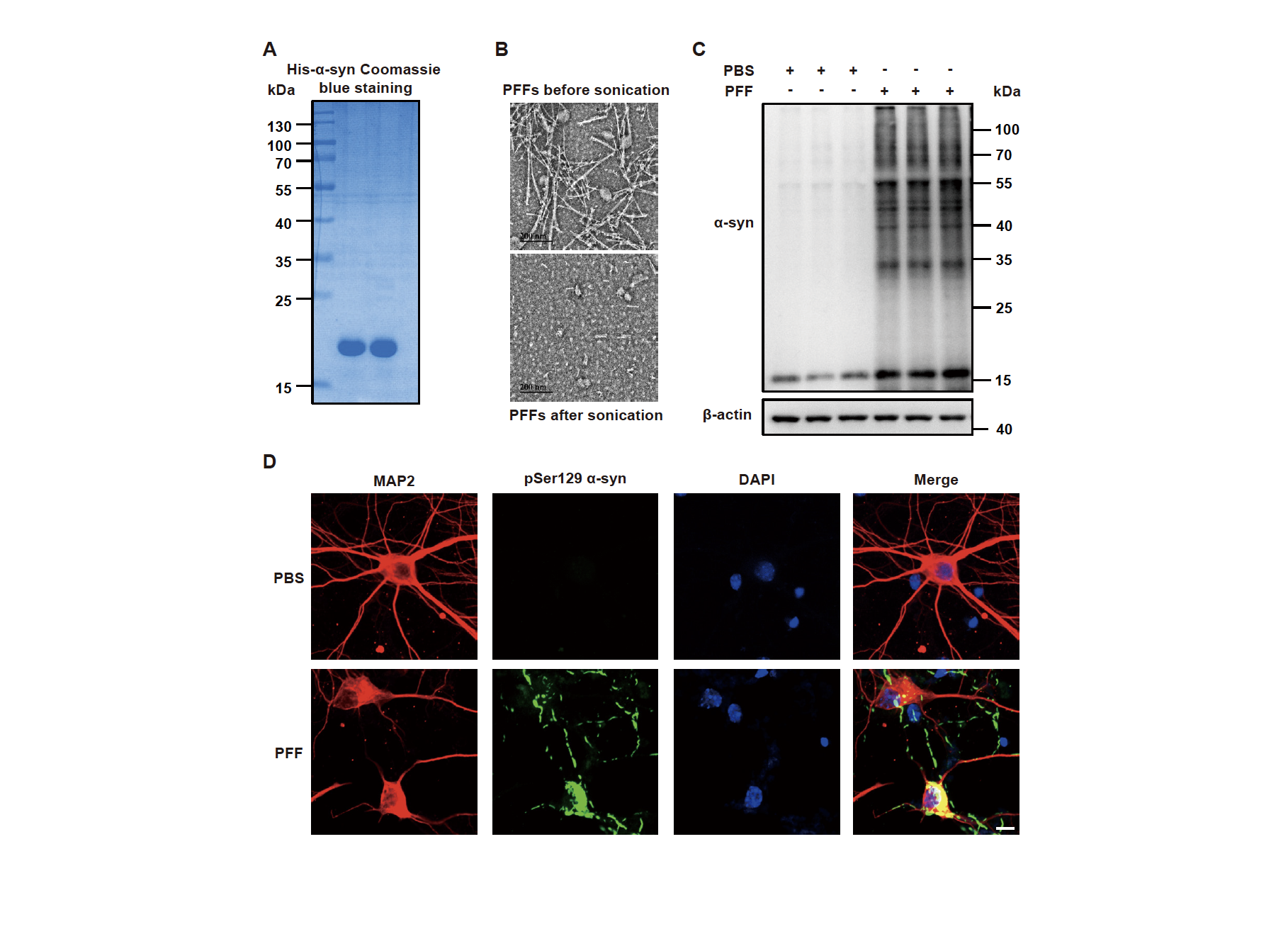
**

**Supplementary Figure 1.** Identification of PFF properties.

**A** Representative images of Coomassie blue staining for the His-α-syn monomer. **B** Representative transmission electron microscopy (TEM) images of PFFs before and after sonication. **C** Primary neurons were incubated with 3 µg/mL PFFs at 7 DIV for 14 days and then processed for analysis. Immunoblots of total α-syn from primary neurons treated with PFF seeds or PBS. **D** Representative images of immunofluorescence staining for pSer129 α-syn and MAP2 in primary neurons treated with PFFs or PBS. Scale bar, 10 μm.

**Supplementary Figure 2**

**
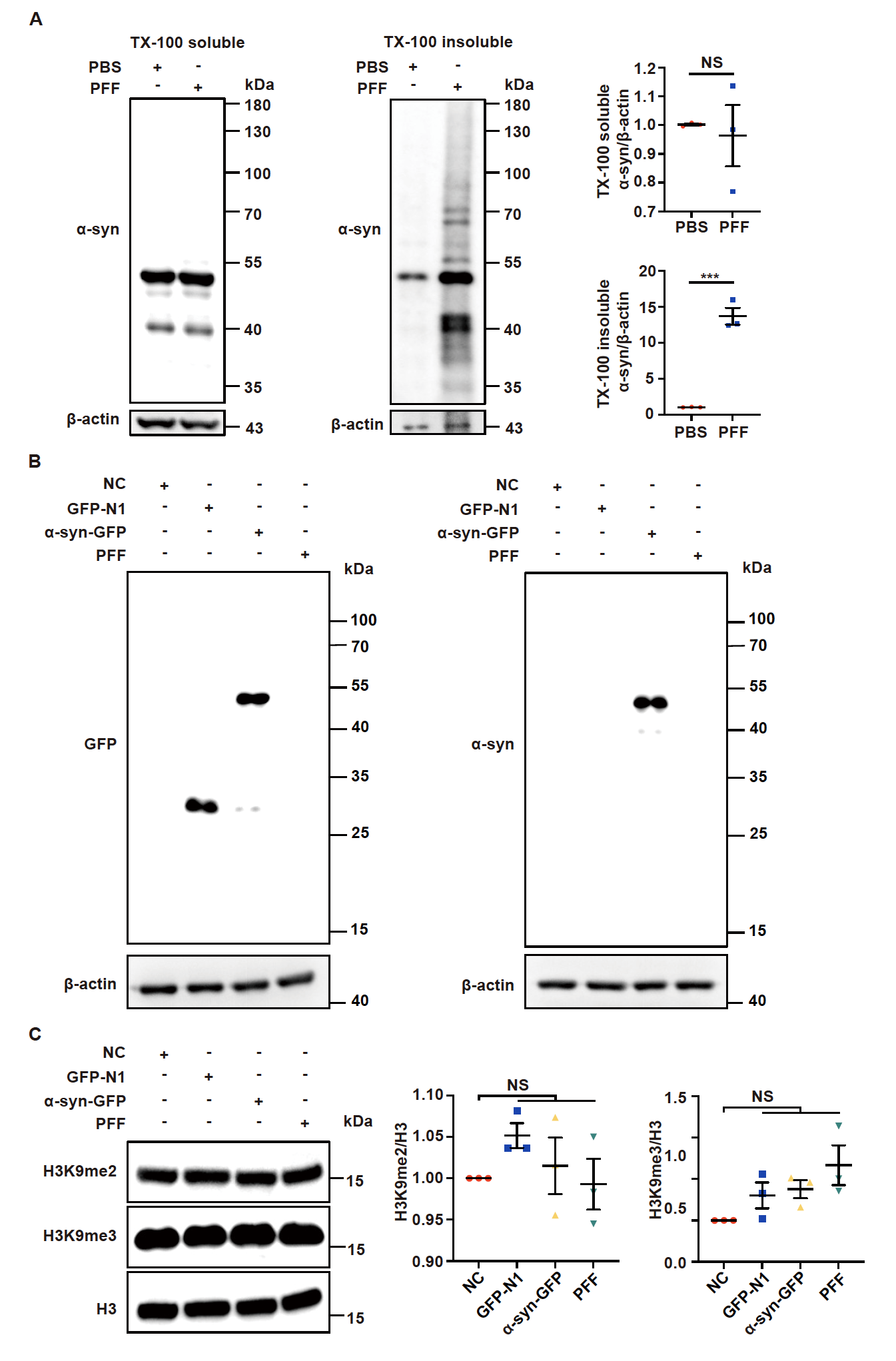
**

**Supplementary Figure 2****.** Alterations in epigenetic regulation depend on the formation of α-syn oligomers in HEK293-α-syn-GFP cells (related to Fig. 1).

**A** Immunoblots and quantitative analysis of Triton X-100 soluble and Triton X-100-insoluble α-syn protein levels in HEK293-α-syn-GFP cells treated with PFFs or PBS (n = 3). PFFs were transfected into HEK293-α-syn-GFP cells at 3 µg/mL for 72 h. **B** Immunoblots of GFP and α-syn from HEK293 cells transfected with GFP-N1, α-syn-GFP, or PFFs at 1 µg/mL for 72 h. **C** Immunoblots and quantitative analysis of H3K9me2 and H3K9me3 from HEK293 cells transfected with GFP-N1, α-syn-GFP, or PFFs at 1 µg/mL for 72 h (n = 3). The *P* values were calculated using unpaired t test in (**A**) or one-way ANOVA in (**C**). ****P* < 0.001, NS indicates not significant. The data are represented as the mean ± SEM.

**Supplementary Figure 3**

**
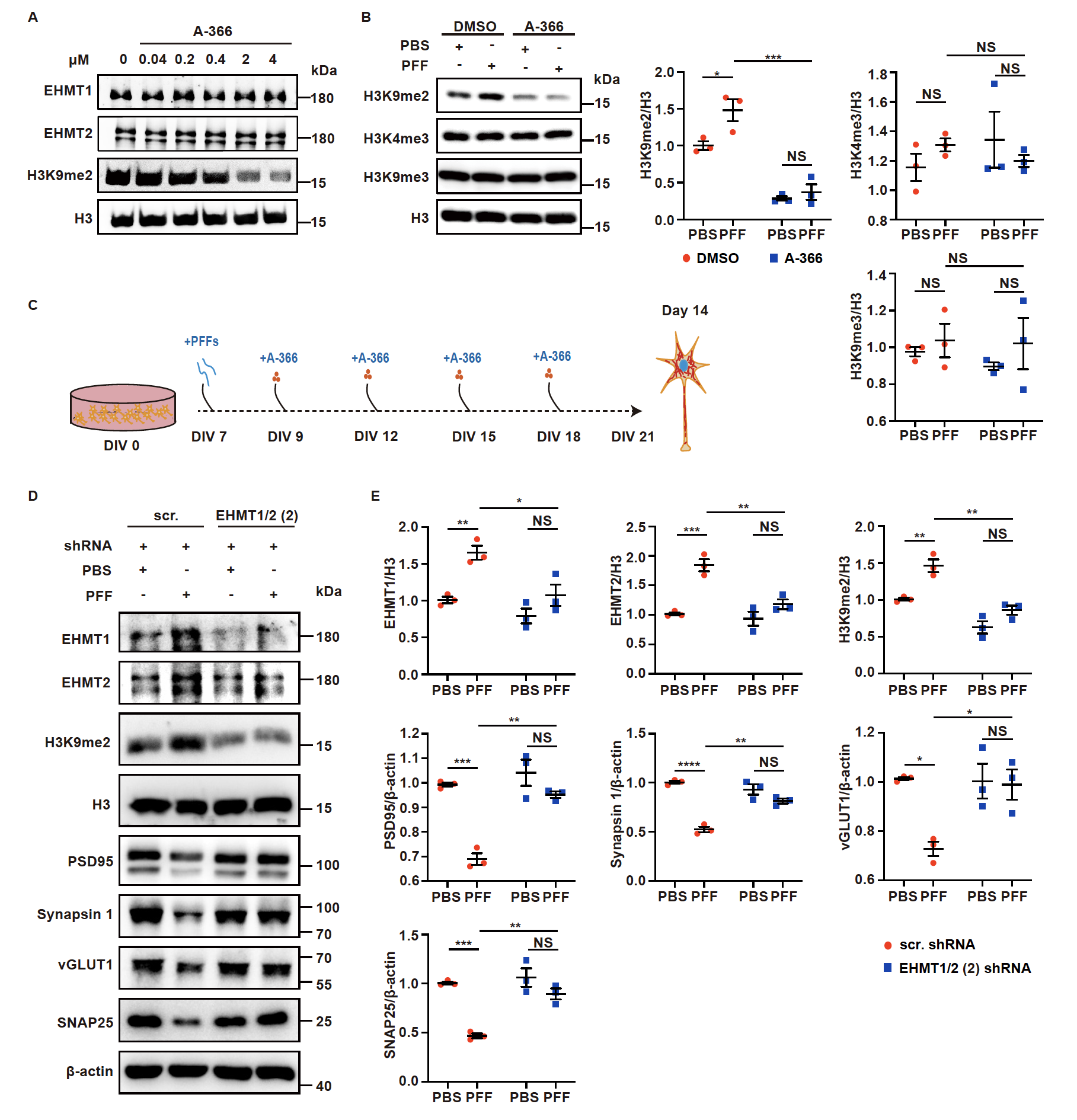
**

**Supplementary Figure 3.** Inhibition of EHMT1/2 rescues the decline in synapse-related proteins by reducing the level of H3K9me2.

**A** Immunoblots of HEK293-α-syn-GFP cells treated with the indicated concentrations of A-366 for 48 h for EHMT1, EHMT2, and H3K9me2. **B** Immunoblots and quantitative analysis of H3K9me2, H3K4me3, and H3K9me3 from HEK293-α-syn-GFP cells treated with A-366 or DMSO and PFFs or PBS (n = 3). A-366 was added at a dose of 2 µM after PFF transfection for 24 h to HEK293-α-syn-GFP cells, which were treated for 48 h. **C** Schematic of the experiments for the time course of the primary culture treatment with PFFs and A-366, with PBS and DMSO serving as controls. **D**-**E** Immunoblots and quantitative analysis of EHMT1, EHMT2, H3K9me2, PSD95, Synapsin 1, vGLUT1, and SNAP25 in primary neurons treated with PFFs or PBS or scr. shRNA or EHMT1/2 (2) shRNA (n = 3). shRNAs were added to primary neurons at 5 DIV. Subsequently, PFFs were added to primary neurons at 7 DIV for 14 days. The *P* values were calculated using two-way ANOVA in (**B**) and (**E**). **P* < 0.05, ****P* < 0.001, *****P* < 0.0001, NS indicates not significant. The data are represented as the mean ± SEM.

**Supplementary Figure 4**

**
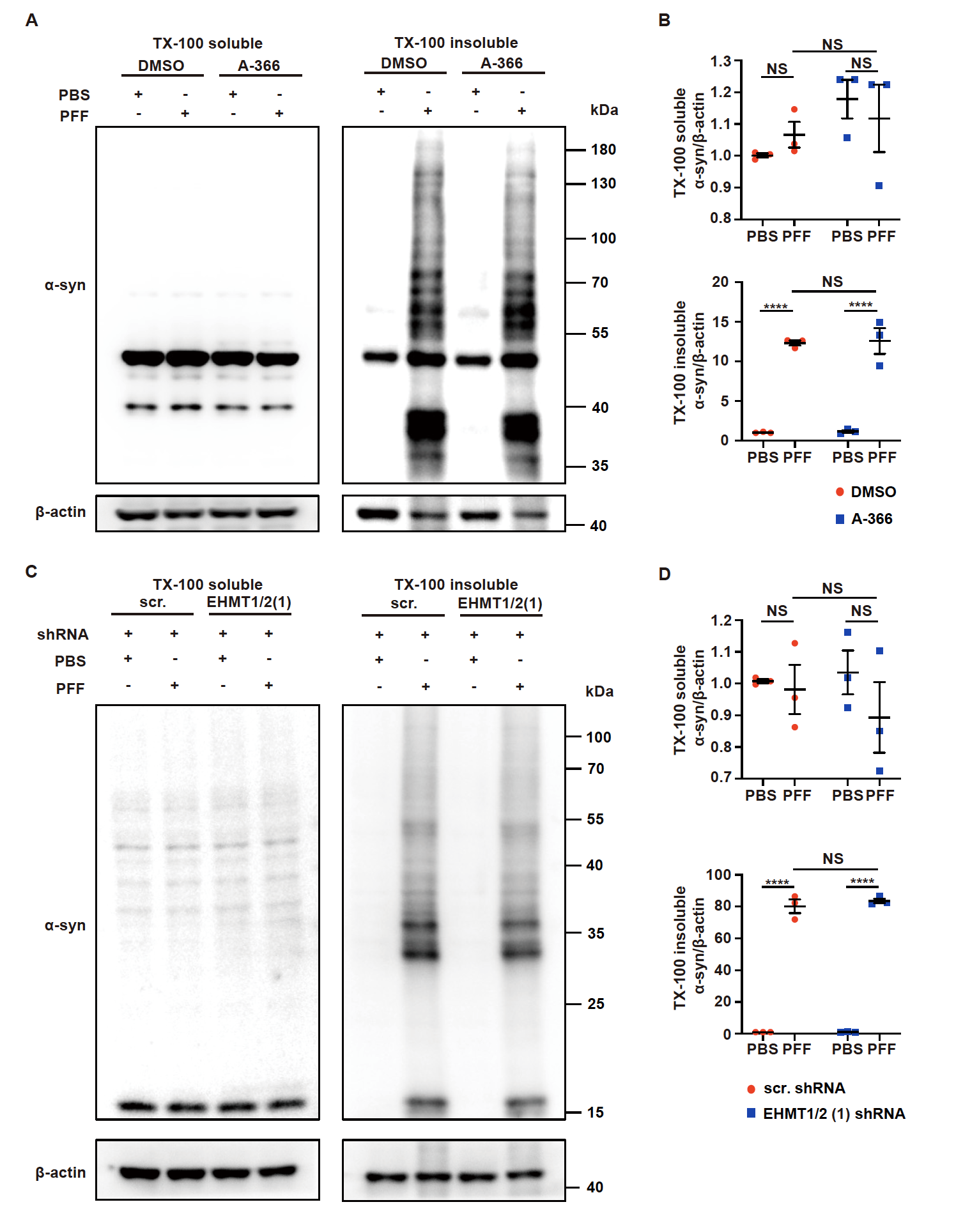
**

**Supplementary Figure 4.** Inhibition of EHMT1/2 has no effect on the formation of α-syn oligomers.

**A**, **B** Immunoblots and quantitative analysis of Triton X-100-soluble and Triton X-100-insoluble α-syn in HEK293-α-syn-GFP cells treated with A-366 or DMSO and PFFs or PBS. (n = 3). A-366 was added at a dose of 2 µM after PFF transfection for 24 h to HEK293-α-syn-GFP cells, which were treated for 48 h. **C**, **D** Immunoblots and quantitative analysis of Triton X-100 soluble and insoluble α-syn in primary neurons treated with PFFs or PBS and scr. shRNA or EHMT1/2 (1) shRNA (n = 3). shRNAs were added to primary neurons at 5 DIV. Subsequently, PFFs were added to primary neurons at 7 DIV for 14 days. The *P* values were calculated using two-way ANOVA in (**B**) and (**D**). *****P* < 0.0001, NS indicates not significant. The data are represented as the mean ± SEM.

**Supplementary Figure 5**

**
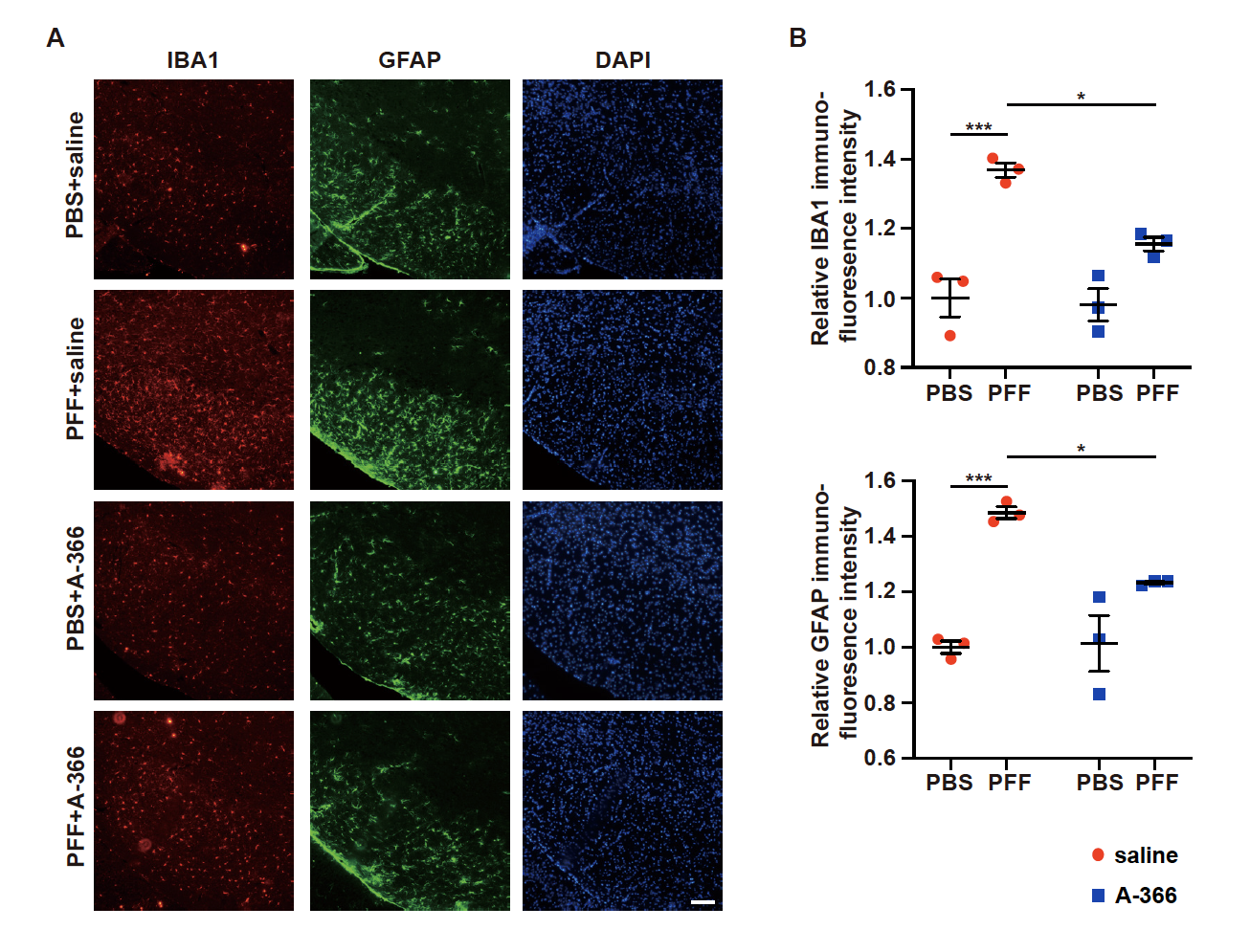
**

**Supplementary Figure 5.** The EHMT1/2 inhibitor A-366 alleviates inflammatory activation in PFF model mice.

Mice were injected with 5 µg of PFFs. Treatment with A-366 was administered according to the schematic diagram depicted in Fig. 6A. **A** Representative images of immunofluorescence staining of IBA1 and GFAP within the midbrain in each group of mice. Scale bar, 100 μm. **B** Quantification of the fluorescence intensity of IBA1 and GFAP (n = 3/group). The *P* values were calculated using two-way ANOVA in (**B**). **P* < 0.05, ****P* < 0.001. The data are represented as the mean ± SEM.

**Supplementary Figure 6**

**
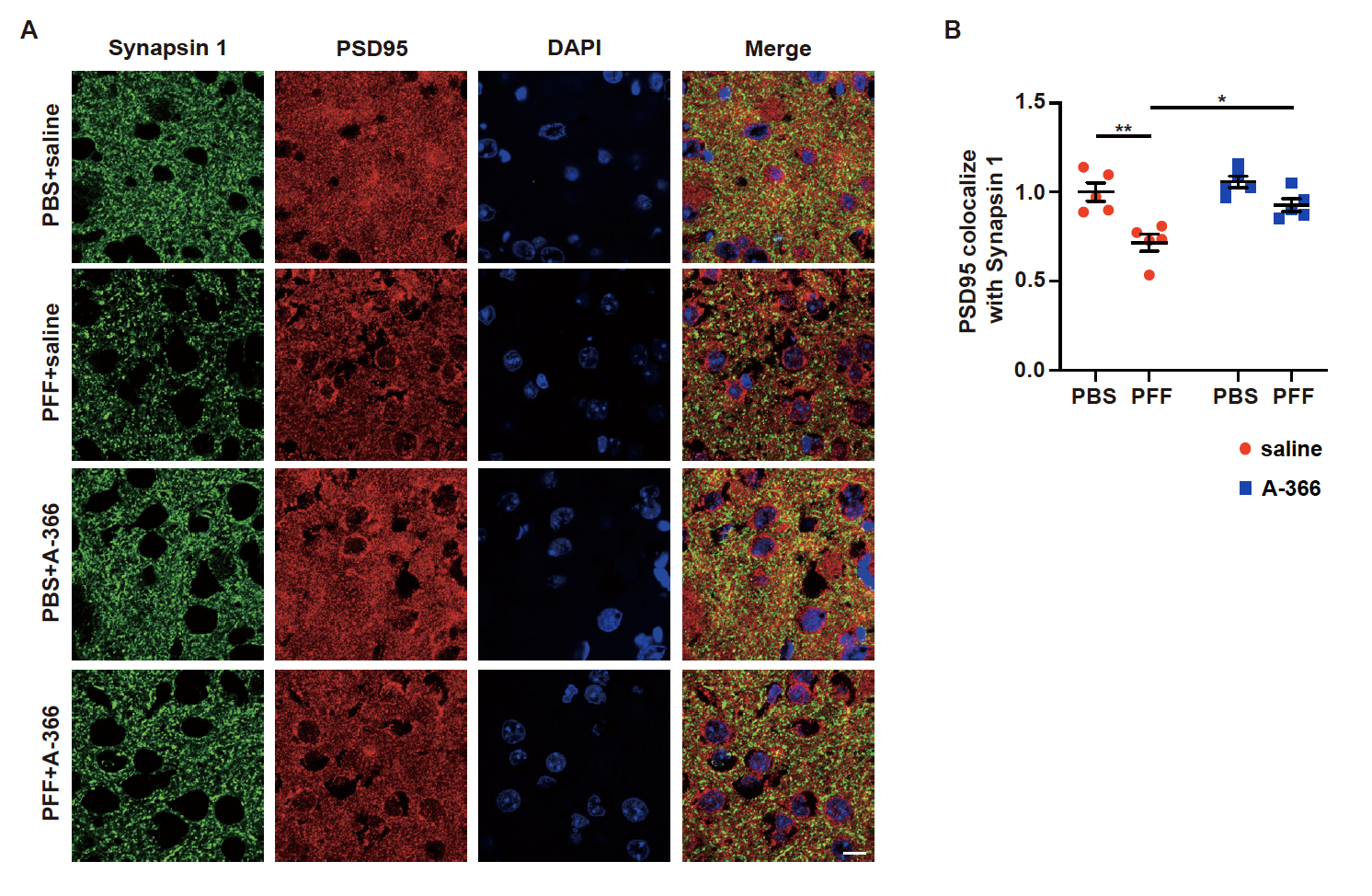
**

**Supplementary Figure 6.** The EHMT1/2 inhibitor A-366 alleviates the decrease in synaptic density reduction in PFF model mice (related to Fig. 8).

Mice were injected with 5 µg of PFFs. Treatment with A-366 was administered according to the schematic diagram depicted in Fig. 6A. **A** Representative images of immunofluorescence staining of Synapsin 1 and PSD95 within the striatum in each group of mice. Scale bar, 10 μm. **B** Quantification of colocalized puncta between presynaptic Synapsin 1 puncta and postsynaptic PSD95 puncta (n = 5/group). The *P* values were calculated using two-way ANOVA in (**B**). **P* < 0.05, ***P* < 0.001. The data are represented as the mean ± SEM.

**Supplementary Table 1. Oligonucleotide primers for PCR were used in this study.**

| Name | Sequence |
| --- | --- |
| *β-actin* | F: GGCTGTATTCCCCTCCATCG  R: CCAGTTGGTAACAATGCCATGT |
| *EHMT1* | F: GAACAGGAGTCTCCCGACAC  R: GGGCTGTCAGTCTTCCCTC |
| *EHMT2* | F: GAAGTCGAAGCTCTAGCTGAAC  R: TGAGGAACCCACACCATTCAC |
| *EZH2* | F: ACTGCTTCCTACATCCCTTC  R: GTGCTGGGTCTGCTACTGT |
| *KMT1a* | F: AACATGCAAGTGGACCCCG  R: GGTATTTTCGGCAAGCCGTT |
| *KMT1b* | F: TTCACAGTGGATGCAGCTCG  R: TGCTTCTCCAGAACCTTTCATT |
| *KMT2C* | F: TGTGAACAAGGGTTCCCGAG  R: GGGAGACAGTGCACATCCAA |
| *PSD95* | F: TGAGATCAGTCATAGCAGCTACT  R: CTTCCTCCCCTAGCAGGTCC |
| *SETD5* | F: GCGTATCACCACTGACCCAA  R: ATCCAGCGCTTCTTACAGGA |
| *SETDB1* | F: ACCCGTCCTAATATGGGTGCT  R: AGCTTTCAGTGTCAGCTGCTT |
| *SNAP25* | F: CAACTGGAACGCATTGAGGAA  R: GGCCACTACTCCATCCTGATTAT |
| *Synapsin 1* | F: AGCTCAACAAATCCCAGTCTCT  R: CGGATGGTCTCAGCTTTCAC |
| *synaptophysin* | F: CAGTTCCGGGTGGTCAAGG  R: ACTCTCCGTCTTGTTGGCAC |
| *Syngr3* | F: ATGGAGGGAGCATCCTTTGG  R: CACCGCAATAGAAAACACCCA |
| *Syntaxin1A* | F: AGAGATCCGGGGCTTTATTGA  R: AATGCTCTTTAGCTTGGAGCG |
| *SYT1* | F: CTGTCACCACTGTTGCGAC  R: GGCAATGGGATTTTATGCAGTTC |
| *SYT2* | F: AGAACCTGGGCAAATTGCAGT  R: CCTAACTCCTGGTATGGCACC |
| *vGLUT1* | F: GGTGGAGGGGGTCACATAC  R: AGATCCCGAAGCTGCCATAGA |
| *VMAT2* | F: ATGGCGGTCAAGGTGCATAC  R: AGTCATGTAATGCTCCCCGTC |

**The quantitative raw data of each figure**

**Figure 1**

**Figure 2**

**Figure 3**

**Figure 4**

**Figure 5**

**Figure 6**

**Figure 7**

**Figure 8**

**Supplementary Figure 2**

**Supplementary Figure 3**

**Supplementary Figure 4**

**Supplementary Figure 5**

**Supplementary Figure 6**
